# Probiotics Stimulate Bone Formation in Obese Mice via Histone Methylations

**DOI:** 10.7150/thno.63749

**Published:** 2021-07-25

**Authors:** Jyotirmaya Behera, Jessica Ison, Michael J. Voor, Neetu Tyagi

**Affiliations:** 1 Bone Biology Laboratory, Department of Physiology, School of Medicine, University of Louisville, Louisville, KY 40202 USA; 2 Department of Orthopaedic Surgery, School of Medicine, University of Louisville, Louisville, KY 40202 USA; 3 Department of Bioengineering, Speed School of Engineering, University of Louisville, Louisville, KY 40202 USA

**Keywords:** gut microbiota, osteoblasts mineralization, histone methylation, mitochondrial biogenesis, bone loss

## Abstract

**Rationale:** Manipulation of the gut microbiome can prevent pathologic bone loss. However, the effects of probiotics on mitochondrial epigenetic remodeling and skeletal homeostasis in the high-fat diet (HFD)-linked obesity remains to be explored. Here, we examined the impact of probiotics supplementation on mitochondrial biogenesis and bone homeostasis through the histone methylation mechanism in HFD fed obese mice.

**Methods:** 16S rRNA gene sequencing was performed to study the microbiota composition in the gut and microbial dysbiosis in obese mouse model. High resolution (microPET/CT) imaging was performed to demonstrate the obese associated colonic inflammation. Obese-associated upregulation of target miRNA in osteoblast was investigated using a microRNA qPCR array. Osteoblastic mitochondrial mass was evaluated using confocal imaging. Overexpression of mitochondrial transcription factor (Tfam) was used to investigate the glycolysis and mitochondrial bioenergetic metabolism using Tfam-transgenic (Tg) mice fed on HFD. The bone formation and mechanical strength was evaluated by microCT analysis and three-point bending analysis.

**Results:** High-resolution imaging (µ-CT) and mechanical testing revealed that probiotics induced a significant increase of trabecular bone volume and bone mechanical strength respectively in obese mice. Probiotics or Indole-3-propionic acid (IPA) treatment directly to obese mice, prevents gut inflammation, and improved osteoblast mineralization. Mechanistically, probiotics treatment increases mitochondrial transcription factor A (Tfam) expression in osteoblasts by promoting Kdm6b/Jmjd3 histone demethylase, which inhibits H3K27me3 epigenetic methylation at the Tfam promoter. Furthermore, Tfam-transgenic (Tg) mice, fed with HFD, did not experience obesity-linked reduction of glucose uptake, mitochondrial biogenesis and mineralization in osteoblasts.

**Conclusions:** These results suggest that the probiotics mediated changes in the gut microbiome and its derived metabolite, IPA are potentially be a novel agent for regulating bone anabolism via the gut-bone axis.

## Introduction

A fat-enriched diet and a sedentary lifestyle are significant environmental factors causing the increased prevalence of obesity and type-2 diabetes 1, 2. Obesity has become a worldwide health problem. It is a state of chronic low-grade inflammation, which increases the risk of diabetes, atherosclerosis, and musculoskeletal disease, including arthritis and bone loss 3-5. Therefore, obesity is associated with reduced lifespan and reduced quality of life 6. The molecular and epigenetic mechanisms governing how obesity negatively regulates multiple organ dysfunctions remain enigmatic. Surprisingly, there are few studies that have examined the negative role of obesity on bone health in animal models. The studies have showed reduced bone mass and quality with altered osteoclast function 7-9. Others have shown that increased osteoclast formation is convoluted with exalted circulating pro-inflammatory cytokines response 10-12.

Recent reports suggest that a moderate increase in plasma concentration of bacterial endotoxin caused bacterial metabolic endotoxemia in mice fed with high-fat diet (HFD) and was responsible for the onset of multiple metabolic diseases 1, 13. Further, prebiotics were to reduce the effect of metabolic endotoxemia and other metabolic disorders in HFD-fed mice 14. These studies suggest that gut microbiota dysbiosis could be responsible for the onset of metabolic disease through metabolic endotoxemia 14. The gut microbiota, an environmental factor residing in the digestive tracts of our bodies, is involved in maintaining gut-barrier integrity 15, intestinal inflammatory responses 16, and in regulating fat storage and physiological homeostasis 17-19. Recent data demonstrate that germ-free (GM) mice have increased net bone formation by reducing levels of osteoclast precursors in the bone marrow (BM) 20. Furthermore, the administration of antibiotics or the probiotic Lactobacillus reuteri prevents bone loss in both young 21 and ovariectomized (OVX) mice, respectively 22. However, the molecular understanding of probiotic (VSL#3)-induced mitigation of gut microbial dysbiosis, inflammation, gut-barrier permeability and metabolic endotoxemia in obese mice remains to be elucidated. Therefore, the present work is warranted to study the preventive action of a probiotic (VSL#3) that mitigates dysbiosis-induced endotoxemia mediated metabolic osteoporosis in experimental obese mice.

Osteoporosis is a major metabolic bone disease and affects >10 million people in the U.S 23. Several factors contribute to osteoporosis, including obesity 23. The prevalence of obesity in the U.S. is much greater and it currently affects >2 billion people worldwide 23. The primary driver of the rise in obesity is attributed to the typical western diet (high in fat, simple carbohydrates, and processed foods) 23. A recent report suggests that an HFD in mice causes reduce bone formation and low mechanical bone quality 24. Indeed, increased osteoclast formation and bone resorption activity, as well as increased number of adipocytes were also observed in HFD-fed mice with associated dysbiosis 25. However, the association of probiotics with restoration of bone mass via gut homeostasis is not known in obese mice. The hypothesis proposed and tested in this work is that, restoring the healthy composition of the gut microbiota through probiotics (VSL#3) supplementation likely plays an essential role in maintaining bone homeostasis in the obese mice.

In the present study, we aimed to restore the healthy gut microbiota composition (or eubiosis) by probiotics treatment (VSL#3) in obese mice and also to reduce the HFD induced gut microbiota dysbiosis, permeability and plasma endotoxemia and its associated skeletal loss. The data demonstrated that probiotics supplementation maintain healthy microbiota composition in gut, maintain gut-barrier integrity and improve osteoblasts mineralization in the bone milieu of HFD-fed obese mice. Mechanistically, osteoblasts differentiation was epigenetically attenuated through hypermethylation of H3K27me3-mark at Tfam promoter. Inhibition of Tfam, due to hypermethylation, exacerbated the glycolysis rate and mitochondrial bioenergetics metabolism and subsequently inhibition of osteogenesis and bone formation, which caused obesity-induced metabolic osteoporosis. Tfam-transgenic (Tg) mice fed with an HFD mostly avoided HFD-induced reduced osteogenesis. These findings uncover previously undefined novel roles of probiotics (VSL#3) to promote bone homeostasis by modulating the gut-bone axis.

## Materials and methods

### Animals and experimental design

The animals were fed standard chow and water *ad libitum*. The animal procedures were carefully reviewed and approved by the Institutional Animal Care and Use Committee (IACUC) at the University of Louisville following the animal care and proper guidelines of the National Institutes of Health. 4-week-old female C57bl6/J mice (WT), Tlr4^fl^ mice (B6(Cg)-Tlr4tm1.1Karp/J) and Osx-GFP::Cre (Osx-Cre) (B6.Cg-Tg(Sp7-tTA,tetO-EGFP/cre)1Amc/J) obtained from the Jackson Laboratories (Bar Harbor, ME). We generated osteoblast specific ablation of Tlr4 in mice (Osx-Tlr4 KO or Tlr4^ObKO^) using Osx-Cre transgenic mice crossed to Tlr4^f/f^ mice 26. 4-week-old Tfam transgenic (Tg) mice were obtained from the Cyagen company and housed in a controlled environment with free access to food and water. The 8-week-old female mice were fed a control non-fat diet (NFD or control WT) or a high-fat diet (HFD) for 8 weeks. The HFD contained 42% fat (corn oil and lard), 28% protein, and <1% carbohydrate. To study the improvement of gut microbiota, we administered the probiotics (VSL#3) to reduce the plasma endotoxemia and promotion of mitochondrial function and bone homeostasis in HFD fed obese mice. The probiotic (VSL#3) was dissolved in sterile drinking water and given through oral gavage in mice at 1x10^9^ CFU every alternative day for 8 weeks.

The following group of mice was used for experimental studies.

- Wild type C57BL/6J (WT)
- Wild-type mice fed with a high-fat diet (HFD)
- Probiotics (VSL#3)-supplemented HFD mice (HFD+Pro)
- Indole-3-propionic acid (IPA)-supplemented HFD mice (HFD+IPA)
- Tlr4^ObKO^ mice fed with HFD (HFD+Tlr4^ObKO^)
- Tfam-Tg mice fed with HFD (Tfam-Tg+HFD)
- Anti-miRNA-138 injected into HFD mice (HFD+anti-miR-138)

### Isolation of mouse primary osteoblasts

Mouse primary osteoblasts were isolated, as previously published protocol 27. After removing the bone marrow (BM) cells from the femoral bone cavities, the bones were cut into small pieces and cultured under an alpha minimum essential medium (α-MEM; Invitrogen) supplemented with 15% heat-inactivated fetal bovine serum (FBS; ATCC), 100 U/mL penicillin/streptomycin and 100 μg/mL ascorbate-2-phosphate (Sigma, St Louis, MO, USA). Cells were cultured for 72 h at 37 °C in a 5% CO_2_ incubation chamber. Then, non-adherent cells were washed out and adherent cells were further cultured. After attaining ten days of culture, the confluent osteoblasts were passaged and maintained for subsequent passaging.

### Osteoblast differentiation and alkaline phosphatase assay

Alkaline phosphatase (ALP) activity and staining were carried out in cultured osteoblasts, as per our previously published protocol stated 28. Briefly, osteoblasts were cultured at a density of 3.5 × 10^5^ cells/well in 12-well culture plates under an osteogenic induction medium (OIM; α-MEM + 15% FBS supplemented with 2 mM β-glycerophosphate, 100 nM dexamethasone, and 50 μg/ml ascorbic acid) for seven days. Then, cells were fixed with 70% ethanol and stained with ALP (Sigma) and the images were photographed using phase-contrast microscopy. The total ALP activity in the cultured osteoblast/well was measured at 405 nm with a microplate reader and presented as nmoles PNPP/mg of protein.

### *In vitro* mineralization assay

*In vitro* mineralization assay was performed as our previously described protocol 29. Briefly, osteoblasts were cultured at a density 3.5 × 10^5^ in 12-well culture plates under OIM for 21 days. Cells were fixed with 70% ethanol for 25 min and stained with 2% Alizarin Red Stain (ARS) (pH 4.2) for 20 min at room temperature. All images were photographed using a phase-contrast microscope. To quantify mineralized nodules, absorbance was read at 510 nm calorimetrically and expressed as an arbitrary unit.

### Osteoclast assay

An osteoclast assay was performed as our previously described protocol 29. Bone marrow (BM) samples were plated at a density of 3.5 × 10^5^ cells/well under α-MEM and were then treated with both RANKL (30 ng/ml) and M-CSF (10 ng/mL) for five days. The media were changed on alternative days. Cells were fixed with 2% paraformaldehyde (PFA) after four days of culture and stained with tartrate-resistant acid phosphatase (TRAP; Sigma). TRAP-positive cells were imaged under a phase-contrast microscope and counted. In addition, plasma TRAP5b activity was determined using an ELISA kit from MyBioSource, as per the manufacturer's instructions.

### Markers of bone turnover

Plasma C-terminal telopeptide of type I collagen (CTX) and N-terminal propeptide of type I procollagen (P1NP) was measured using an ELISA kit from MyBioSource, as per the manufacturer's instructions.

### RNA extraction from microbial colon contents

Bacterial RNAs were isolated from colonic contents using the RNeasy Mini Kit (Qiagen, Hilden, Germany), as per the manufacturer's instructions. Briefly, colonic contents were homogenized using sterile mortar and pestle for 2 min and collected in a sterile microcentrifuge tube containing a 750 µL QIAzol lysis reagent. Then, samples were vortexed for 15 s following the addition of 150 µL chloroform: isoamyl alcohol 24:1) and allowed to stand for 2-3 min at room temperature. Then, the samples were centrifuged at 10,000*g* for 5 min at 4 °C, and pellets were washed with ice-cold ethanol. Following centrifugation, supernatants were removed, and pellets dried at room temperature for 10 min. We then resuspended the RNA pellet in 50 μL of RNase-free water and stored at -80 ^o^C until use.

### Microbiota analysis by 16S rRNA Sequencing

Eight-week-old female C57bl6/J mice (WT) were obtained from the Jackson Laboratories (Bar Harbor, ME). The mice were fed a control non-fat diet (NFD or control WT) or a high-fat diet (HFD) for 8 weeks. In the separate cohort of the HFD group, mice were administered the probiotics (VSL#3) at 1x10^9^ CFU for 8 weeks on alternative days. After 8 weeks of feeding, fecal pellets were collected from each group of mice, and DNA was extracted using the MoBio DNA isolation kit. The V4 region of the 16S genes of bacterial genomes will be amplified using the methods of Tyagi et al. 30, 31. Amplicons were sequenced on an Illumina MiSeq instrument at the University of British Columbia (Microbiome Insights Inc., Canada). Analysis of the sequencing reads of the samples was performed using the standard methodology for microbiome analysis. Briefly, the raw sequence was processed via QIIME. Further, phylogeny and diversity analyses were done using the QIIME and MOTHUR pipelines.

### Mass spectrometry analysis of microbial metabolite analysis

Fecal samples from colonic contents were harvested from experimental mice as previously described 32 to measure tryptophan metabolites. Indole-3-acetic acid (IA), indole-3-propionate (IPA), 5-hydroxy 5-hydroxyindoleacetic acid (5-HIAA) levels were measured using an ELISA kit from MyBioSource, and Eagle Biosciences, Inc. as per the manufacturer's instructions.

### *In vivo* indole-3-propionate treatment

For indole-3-propionate (IPA) administration, HFD mice were supplemented with indole-3-propionate (20 mg/kg/day) 32 or vehicle by oral gavage once daily (weekly five days) for 8 weeks (total 40 gavages). Gut microbiota changes were verified by quantitative real-time PCR (qPCR) using universal primer 16S rRNA. At the end of the indole-3-proponate administration, the HFD+IPA mice group were used for downstream *in vivo* experiments and tissue was harvested for analysis and use with *in vitro* experiments.

### Intestinal permeability assay

Gut-barrier function was evaluated in the experimental mice by measuring *in vivo* intestinal permeability to 4,000-Da fluorescent-labeled dextran (Sigma-Aldrich), as previously described 33. Briefly, 12 h-fasted experimental mice were administered fluorescein isothiocyanate-labeled 4.4-kDa dextran (0.5 ml of 22 mg/ml) by oral gavage. After 4 h of administration, blood was collected by terminal cardiac puncture. The plasma dextran concentration was analyzed with a fluorescence spectrophotometer standard with excitation at 485 nm and emission at 530 nm. Further, dextran concentration was calculated by comparing samples with a serial dilution of known standards.

### Colon histology

Following the last treatment of probiotics or IPA, colons were collected and fixed in 10% neutral buffered formalin for 24 h. Subsequently, colon samples were dehydrated, embedded in paraffin, sectioned at six μm, and stained with hematoxylin and eosin (H&E). H&E-stained colon sections were imaged using phase-contrast microscopy and then scored as described previously 34. Briefly, histopathological lesions were evaluated using the disease activity index (DAI), as previously described 34. The DAI includes the degree of mucosal damage (0: none, 1: mucous layer, 2: submucosa, 3: muscularis and serosa), and crypt damage (0: none, 1: basal, 1/3: damaged, 2: basal, 2/3: damaged, 3: entire crypt damaged, 4: epithelium lost). All the parameters were summed. A total colon score was calculated by adding all three colon sections.

### *In vivo* PET/CT imaging of Intestinal inflammation and glucose uptake in bone

Mice in the intestinal inflammation study were assigned into three groups: WT control, HFD, HFD+IPA, and HFD+Pro mice. All experimental groups of mice underwent overnight (or at least 12 h) fasting before Positron emission tomography (PET)/computed tomography (CT) scanning but had free access to water. Following the determination of body weights, mice were anesthetized using vaporized isoflurane (4% for induction; 2.5% for maintenance) and sterile normal saline (0.1 ml) was injected subcutaneously to ensure adequate hydration. All animals received 200 μl of ProSense 680 Fluorescent Imaging Agent (PerkinElmer, cat. No: NEV10001EX) by rectal injection. After 30 min, these mice received 100 μL of MD-Gastroview as a contrast agent rectally via a 3.5F catheter. Subsequently, all animals were CT scanned for 10 min, followed by a PET scan for 20 min using the Inveon small-animal PET/CT imaging system (Siemens, Munich, Germany) in the radiology facility of the University of Louisville.

Similarly, to determine the uptake of glucose in femoral bone, separate groups of mice were administered ^18^F-FDG (8.65±2.7 MBq) via intraperitoneal (i.p) injection as previously described 35. At 45 min of post-FDG injection, mice were re-anesthetized and scanned using an Inveon small-animal PET/CT imaging system (Siemens Healthcare Molecular Imaging, USA). Mouse images were recorded, ^12^F-AV and ^18^FDG standardized uptake values were analyzed blindly (R.R.S.) using Inveon Research Workplace software version 1.5 (Siemens). The images of mice were generated by OsiriX Imaging software version 3.9.4 (Pixmeo, Bernex, Switzerland).

### Glucose Tolerance test

Glucose tolerance test (GTT) was performed as previously described 36. Mice were overnight fasted and then injected with glucose (1 g/kg b.wt), intraperitoneally. Following injection, glucose levels were detected in blood obtained from the tail tip of mice at the interval of 15, 30, 60, and 120 min using an ACCU-CHEK Advantage Glucometer (Roche Diagnostics, Laval, QC, Canada).

### Biochemical analyses

Plasma endotoxin levels were examined using the Limulus Amebocyte Lysate kit from Sigma-Aldrich, according to the manufacturer's instructions. Plasma glucose concentration was determined in 10 µL plasma using a Glucose (GO) Assay kit (Sigma-Aldrich), according to the manufacturer's instructions. Plasma insulin concentration was determined in 10 µL plasma using Mouse Ins1 / Insulin-1 ELISA Kit (Sigma-Aldrich), according to the manufacturer's instructions.

### microRNA (miRNA) profiling by RT2 miRNA PCR array

Total RNA was isolated from osteoblasts using the miRNeasy Mini Kit (Qiagen, USA), according to manufacturer's instructions, and RNA was converted to cDNA using the miScript II RT kit (Qiagen). The RT^2^-qPCR array was performed on 96-well plates (MIMM-001Z, miScript miRNA PCR Array Mouse miFinder, Qiagen), according to manufacturer's instructions. For individual microRNA expression, cDNA was used for amplification using a miScript SYBR Green PCR kit. The miRNAs amplification was performed in Stratagene Mx3000p (Agilent Technologies, Santa Clara, CA, USA), the Ct values were determined, and the results are expressed in fold change in expression.

### Real-time quantitative PCR

Total RNA from femoral bone tissues was prepared using the TRIzol reagent (Thermo Fisher Scientific, USA), according to the manufacturer's instructions. Using 1 µg of isolated total RNA, cDNA was synthesized using a reverse transcription kit (Promega, Madison, WI). Quantitative PCR (qPCR) was performed to amplify individual genes using LightCycler® 96 System - Roche Life Science and the accompanying software. The PCR conditions were performed with the following programs: 1.5 min at 50 °C, 8 min at 95 °C followed by 35 cycles of two-step PCR denaturation at 95 °C for 18 s and annealing extension at 60 °C for 55 s. Each sample contained 10 ng cDNA in 1x SYBRGreen PCR Master Mix (Roche) and 10 pmol/l of each primer in a final volume of 20 µL. The relative amount of each studied mRNA was normalized to GAPDH levels as a housekeeping gene, and the data were expressed according to the 2^-ΔΔCT^ method. Primer sequences for the targeted mouse genes are presented in Supplementary Table S1.

### *In Silico* Analysis

To predict potential targets of miR-138 at the mouse mRNA region,* in silico* analyses were performed using Targetscan (http://www.targetscan.org/), miRcode (http://www.mircode.org/index.php), and microRNA.org (http://www.microrna.org/microrna/ home.do).

### AntagomiR-138 injection and overexpression of Kdm6b* in vivo* and *in vitro*

To investigate the role of miR-138 and JMJD3/Kdm6b function in HFD fed mice, the miRNA inhibitors or synthetic antagomiRs against the miR-138 (miR-138 inhibitor: AGCUGGUGUUGUGAAUCAGGCCG) were purchased (Sigma, USA) and delivered via intravenous tail-vein injections (2 µg per injection) 2-times per week, up to 4-weeks (total 8 injections). The antagomiRs are stable for up to 2-weeks and inhibit the target miRNA via degradation 37. Moreover, to overexpress Jmjd3 expression, osteoblasts were transfected pcDNA3.1-JMJD3 plasmid (100 ng, Addgene) using a Lipofectamine 3000 reagent (Invitrogen, Carlsbad, CA, USA). After 48 h of transfection in osteoblasts, Jmjd3 overexpression was confirmed with qPCR analysis.

### KDM6b-UTR-3' Luciferase assay

Kdm6b 3'UTR Lenti-reporter-Luc Vector was purchased (Cat. No: 254870840195, ABM, Richmont, Canada) and transfected to cultured osteoblasts under serum-free medium, using Lipofectamine 3000 (Invitrogen, Carlsbad, CA, USA). Cell lysates were prepared after 48 h following transfection, and luciferase activity was determined using the Dual-Luciferase Reporter Assay (Promega, Madison, WI, USA). Results were further normalized to the Renilla luciferase.

### RNA Immunoprecipitation (RIP) Assay

RIP assay was performed as previously described 43. The Magna RIP™ RNA-Binding Protein Immunoprecipitation Kit (Millipore) was used for the RIP assay to study the presence of miRNA-138 on the target protein. Briefly, about 3.5 × 10^6^ of osteoblasts were cultured under α-MEM. Following confluency, cells were lysed with RIPA lysis buffer and then incubated with magnetic beads conjugated with the anti-H3K27me3 antibody or rabbit IgG control. The immunoprecipitated RNAs were purified and subjected to the RT-qPCR assay to evaluate the enrichment degree of miRNA-138.

### Chromatin Immunoprecipitation (ChIP) Assay

A chromatin immunoprecipitation assay was performed as previously described 36. Chromatin preparation was done from the osteoblasts using the ChromaFlash Chromatin Extraction Kit (Epigentek, Farmingdale, NY, USA), according to the manufacturer's instructions. Briefly, the prepared chromatin was immunoprecipitated with anti-KDM6b antibody-ChIP grade (ab85392, Abcam) or anti-Histone H3 (tri methyl K27) antibody- ChIP Grade (ab6002, Abcam) overnight. It was amplified by PCR reaction using LightCycler® FastStart DNA Master SYBR Green I (Roche Life Science) and PCR instrumentation LightCycler® 96 Instrument (Roche Life Science). Primers were designed for the Tfam promoter. The primer pairs used to amplify sequences surrounding predicted KDM6b or H3K27me3 binding sites at the Tfam locus are presented in Table S1.

### H3-K27 specific histone demethylase, Jmjd3 demethylase activity assay

Nuclear extracts from osteoblasts were prepared with the EpiQuik Nuclear Extraction Kit (Epigentek, Farmingdale, NY) according to the manufacturer's instructions. Further, the activation of Jmjd3 in osteoblasts were assessed with the Epigenase JMJD3/UTX Demethylase Activity/Inhibition Assay Kit (Colorimetric) (Catalogue no: P-3084-48, Epigentek), according to the manufacturer's instructions.

### Western blot analysis

Both cultured osteoblasts and femoral bone tissue were homogenized separately in an ice-cold RIPA buffer containing PMSF (1 mM) and protease inhibitor cocktails (1 μl/ml of lysis buffer; Sigma Aldrich, St. Louis, MO, USA) 28. The extracted proteins were centrifuged at 12,000×g for 7 min at 4 °C. The supernatant was collected and stored at -80 °C until use. The collected supernatants (50 μg) were loaded on a sodium dodecyl sulfate-polyacrylamide gel electrophoresis (SDS-PAGE) and run at a constant 110 V. Separated proteins in the gels were transferred using an electrotransfer apparatus (Bio-Rad) to polyvinylidene fluoride (PVDF)-membranes. Then, membranes were blocked with 5% nonfat dry milk in TBS-T solution for 1-1.5 h, followed by incubation in the desired primary antibody at 4 °C overnight. After washing with TBS-T for 7 min each for three times, the membranes were incubated with a secondary antibody [conjugated with horseradish peroxidase] for 90 min at room temperature with constant shaking. The membranes were further developed with the ECL Western blotting detection system (GE Healthcare, Piscataway, NJ, USA) and images were taken using the ChemiDoc XRS+ gel documentation system (Bio-Rad). Band density was normalized and calculated with a housekeeping control (GAPDH protein) using Image Lab densitometry software (Bio-Rad) and expressed as fold change in protein expression.

### Dynamic histomorphometric analysis

Bone histomorphometric analysis was carried out as our previously published protocol states 28. The excised bone femurs were collected and fixed in 10% neutral buffered formalin overnight and decalcified in 0.5 M ethylenediamine tetraacetic acid (EDTA) for 30 days. Then, decalcified femurs were embedded in paraffin wax and cut into five μm slices using a Leica RM2125 RTS microtome. The femur slices were stained with H&E solutions (Sigma) and the images were photographed using a phase-contrast microscope with 10× magnification. Five representative images were analyzed, and the results were presented as the percentage (%) of trabecular bone per total bone area.

### 3-Point bending test of bone

Bone mechanical quality was carried out as our previously described protocol 28, 29. Femoral bones were dissected and fixed in 10% neutral buffered formalin for two days and stored at -80 °C in 70% ethanol until use. The 3-point bending test was performed to examine the femoral bone mechanical quality or cortical bone strength. All bone samples were tested under a load applied at a constant rate of 20 mm/min. The following parameters were measured including maximum load (ultimate strength) and stiffness (slope of the linear portion of the curve representing elastic deformation) by using the Bone Strength Tester servohydraulic test system (Model 858 Mini Bionix, MTS Systems Corporation, Eden Prairie, MN).

### MicroCT analysis of bone

The excised femoral bones were fixed in 10% neutral buffered formalin for two days and stored at -80 °C under 70% ethanol until use. Briefly, the femur bones were scanned using the SkyScan 1174 μ-CT scanner (Aartselaar, Belgium). Bone reconstructions were then carried out using Sky Scan Nrecon software. 3-D images of the trabecular region of femoral bones were drawn by OsteoQuant software by selecting each region of interest (ROI). The trabecular bone volume/tissue volume BV/TV (%), trabecular number (Tb.N) (mm^-1^), trabecular separation (Tb.Sp) (μm) and trabecular thickness (Tb.Th) (μm) were calculated.

### Statistical analysis

Data analyses and graphical presentations were performed with GraphPad Prism, version 8.0.3 (GraphPad Software, Inc., La Jolla, CA). All data are reported as mean ± SEM. Normality was determined by the D'Agostino-Pearson omnibus test. The experimental groups were compared by one-way analysis of variance (ANOVA) in combination with Tukey's multiple comparison test. The significance of differences between the two groups was determined using Two-tailed, unpaired Student's t-test. p< 0.05 was considered statistically significant.

## Results

### Probiotics supplementation increases indole-3-propionate levels and prevents gut inflammation, and permeability in obese mice

**Figure 1:**
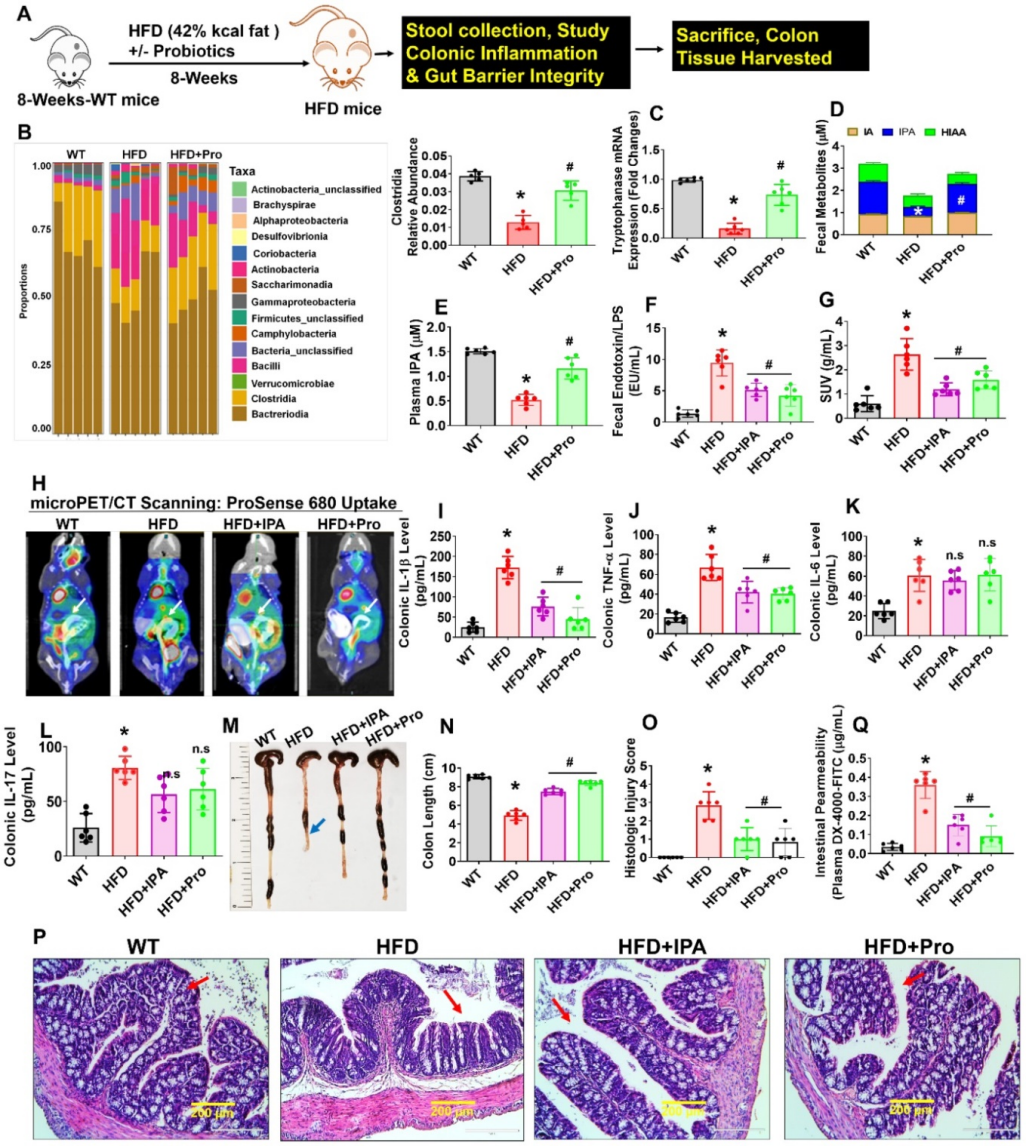
** Probiotics-treatment increases IPA-producing lactobacilli and prevents intestinal inflammation and permeability in HFD fed obese mice. (A)** 8-weeks-old C57BL/6 mice were fed on HFD (42%) with or without probiotics (VSL#3) treatments for periods of 8-weeks. Following treatments, the samples were harvested for further experiments. **(B)** The relative abundance of bacterial taxa at the class level within fecal pellets collected from mice treated with probiotics or vehicle control for 8 weeks. *p < 0.0001 compared with the WT control, #p < 0.0001 compared with the HFD. **(C)** qPCR transcript expression of tryptophanase in the colonic fecal contents in mice. *p < 0.0001 compared with the WT control, #p < 0.0001 compared with the HFD. **(D)** Tryptophan metabolites concentrations (IA, I3A, IPA, 5-HIAA) in fecal contents of the colon of mice administered probiotics or vehicle control for 8 weeks. *p < 0.0001 compared with the WT control, #p < 0.0001 compared with the HFD (for IPA). **(E)** The plasma IPA level was measured. *p < 0.0001 compared with the WT control, #p < 0.0001 compared with the HFD. **(F)** Endotoxin levels were measured in the fecal contents of mice. *p < 0.0001 compared with the WT control, #p < 0.0001 (HFD+Pro), #p < 0.0003 (HFD+IPA) compared with the HFD. **(G-H)** Representative microPET/CT images of inflammation of experimental mice acquired with ^18^F-AV uptake in the intestine. Images were acquired 30 min after injection of ^18^F-AV and are displayed as [18F] AV SUVmeanvalues. *p < 0.0001 compared with the WT control, #p < 0.0022 (HFD+Pro), #p < 0.0001 (HFD+IPA) compared with the HFD. **(I-L)** Colonic inflammatory cytokines (IL-1, TNF-α, IL-6 and IL-17) levels were estimated using ELISA. *p < 0.0001 compared with the WT control, #p < 0.0001 compared with the HFD for figure 1I. *p < 0.0001 compared with the WT control, #p < 0.0004 (HFD+Pro), #p < 0.0009 (HFD+IPA) compared with the HFD for figure 1J. **(M-N)** Representative images of the colon of the experimental mice. The bar graph represents the length of the colon tissue. *p < 0.0001 compared with the WT control, #p < 0.0001 compared with the HFD. **(O-P)** Representative images of the H&E-stained colonic tissue section from the four experimental mice group **(O)**. The histologic inflammation/injury scores of the colon were examined in the experimental groups **(P)**. *p < 0.0001 compared with the WT control, #p < 0.0001 (HFD+Pro), #p < 0.0003 (HFD+IPA) compared with the HFD. *Scale bar: 200 µm.* **(Q)** Measurement of intestinal permeability in WT, HFD, HFD+IPA, and HFD+Pro mice. *p < 0.0001 compared with the WT control, #p < 0.0001 compared with the HFD. All the data (Figure-1) are analyzed by one-way ANOVA followed by a Tukey's multiple comparisons test. n = 6 mice per group. All data are expressed as mean± s.e.m.

Eight-week-old female mice were conventionally raised and supplemented by oral gavage with 1×10^9^ CFU probiotics (VSL#3) or vehicle control to HFD fed obese mice for 8 weeks (Figure 1A). We collected fecal samples from the various experimental groups and performed 16S rRNA taxonomic profiling to understand changes in microbial community structure during obesity. As a measure of gut microbial diversity, we identified that clostridia phyla were significantly lowered in HFD group compared to WT control. While Probiotics supplementation in the HFD mice group resulted in a change in gut microbial diversity in the colon of the intestinal lumen, suggesting an expansion in the proportion of tryptophan metabolites producing *clostridia* (Figure 1B). Next, we investigate the expression level of bacterial gene coding for tryptophanase enzyme, which is involved in tryptophan metabolites; indole and its derivatives, indole-3-proponate (IPA) production by tryptophan-utilizing bacteria 30, 37 in the gut. The qPCR analysis suggested that tryptophanase enzyme mRNA transcript expression was significantly downregulated in HFD group compared to WT. However, probiotic treatment enhanced its mRNA expression in HFD+Pro group compared to HFD alone (Figure 1C). Using ELISA, reduced levels of IPA were detected in fecal samples of HFD group compared to WT control, whereas the level of IPA was restored to normal in the HFD+Pro mice group compared to HFD mice (Figure. 1D). However, indole-3-acetic acid (IA), and 5-hydroxy 5-hydroxyindoleacetic acid (5-HIAA) levels were not changed among the groups (Figure 1D). Consistent with these findings, a reduced level of IPA was also detected in plasma of HFD group compared to WT control. However, probiotics treatment restored the plasma level of IPA in HFD+Pro group compared to HFD group (Figure 1E). In contrast, both fecal and plasma level of endotoxin was significantly increased in HFD group compared to WT control. Probiotic or IPA supplementation mitigated the above effects in HFD+Pro and HFD+IPA group respectively compared to HFD mice alone (Figure 1F, Figure S1A).

To study further how probiotics treatment, or IPA injection directly to HFD mice, prevented gut inflammation, we administered ProSense 680 Fluorescent Imaging Agent via rectal route 30 min before microPET/CT scanning. ProSense 680 Fluorescent Imaging Agent uptake allowed visualization of increased colon tissue associated with inflammation in HFD mice compared to WT control (White arrow, Figure 1G-H). Probiotics or IPA supplementation prevented colonic inflammation in HFD+Pro or HFD+IPA mice groups respectively compared to HFD mice group (Figure 1G-H). We also tested colonic cytokine (IL-1, TNF-α, IL-6, IL-17) levels in homogenized tissue extract of experimental mice. The data demonstrate that colonic levels of IL-1β, TNF-α cytokines, but not IL-6 and IL-17 were significantly increased in HFD group compared to WT control. However, probiotics or IPA supplementation substantially reduced the colonic IL-1β, TNF-α cytokine levels, but not IL-6 and IL-17 in HFD+pro or HFD+IPA mice compared to HFD mice alone (Figure 1I-L). Systemic plasma cytokines (IL-1β, TNF-α) levels were also significantly increased in HFD group compared to WT control, whereas its levels were mitigated in HFD+Pro or HFD+IPA mice (Figure S1B-C).

Recent studies have shown that the colon of animals fed an HFD is shorter in length than those of WT control animals 38. In our study, probiotics or IPA supplementation led to a significant increase in the intestinal length (Figure 1M-N, blue arrow). To test whether probiotics or IPA functionally contributed to preventing colonic tissue architectural damage, we examined the histological analysis of colonic tissue. Notably, HFD mice had significantly higher mucosal tissue damage. However, probiotics, or IPA, treated mice had significantly lower mucosal tissue damage and reformation of microvilli architecture in HFD+pro or HFD+IPA mice (Figure 1O-P, red arrow). Using fluorescein isothiocyanate (FITC)-Dextran assay, we determined intestinal permeability and found that HFD mice were associated with increased intestinal permeability compared to WT control. However, the above changes were mitigated in HFD+Pro or HFD+IPA mice (Figure 1Q). Consistent with increased permeability in the HFD colon, mRNA transcript expressions of the tight junction (TJ) protein genes (ZO-1, Occludin and Claudin-5) in colon tissue were also significantly lower in the HFD mice compared to WT control mice. Treatment with probiotics or IPA restored the colon to the normal condition (Figure S1D-F). Thus, increased inflammation and leakiness of the gut is a consequence of HFD. These data conclude that HFD induced obesity is associated with gut microbial dysbiosis, intestinal inflammation, and permeability, and probiotics or IPA treatment mitigates the above obesity-related changes (Figure 1).

### Probiotics treatment prevented obesity-induced suppression of Tfam expression in osteoblast via miRNA-138-JMJD3-H3K27me3 epigenetic cascade

**Figure 2:**
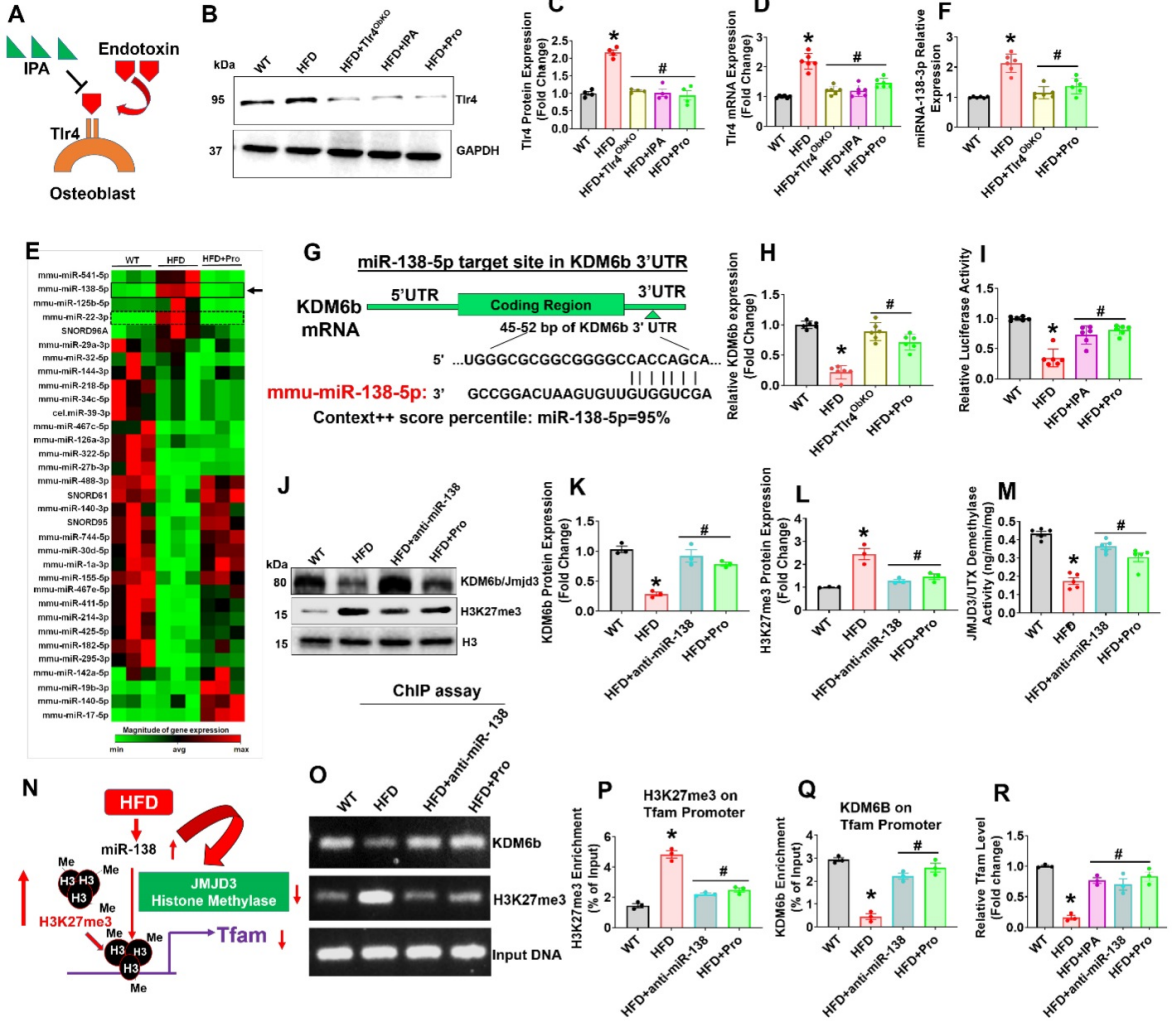
** Probiotics treatment promoted Tfam expression in osteoblast via mitigating Tlr4-miRNA-138-H3K27me3 epigenetic cascade. (A)** Proposed mechanism of action of inhibitory activity of IPA on Tlr4 in osteoblast and prevents the endotoxin induced activation of Tlr4 action. **(B-C)** Tlr4 protein expression was studied using western blot analysis. *p < 0.0001 compared with the WT control, #p < 0.0001 compared with the HFD. **(D)** mRNA transcript expression of Tlr4 using qPCR. *p < 0.0001 compared with the WT control, #p < 0.0001 compared with the HFD. **(E)** Heat map of miRNA PCR Array shows differentially expressed miRNAs in osteoblast of experimental mice. **(F)** qRT-PCR validation of miRNA-138 expression in osteoblast of experimental mice. *p < 0.0001 compared with the WT control, #p < 0.0001 compared with the HFD. **(G)** Schematic representation of the miR-138 binding sites in histone demethylase, Kdm6b/Jmjd3. **(H)** qRT-PCR analysis of Kdm6b/Jmjd3 expression level in osteoblast. *p < 0.0001 compared with the WT control, #p ≤ 0.0001 compared with the HFD. **(I)** The luciferase activity was examined in osteoblast that were transfected with reporters containing Kdm6b 3' UTR reporter vector. *p < 0.0001 compared with the WT control, #p ≤ 0.0001 compared with the HFD. **(J-K, L)** Protein expression of Kdm6b, and H3K27me3 was performed by western blot analysis. *p < 0.0001 compared with the WT control, #p < 0.0004 (HFD+anti-miR-138), #p < 0.002 (HFD+Pro) compared with the HFD for figure 2K., *p = 0.0004 compared with the WT control, #p < 0.0018 (HFD+anti-miR-138), #p < 0.0052 (HFD+Pro) compared with the HFD for figure 1L. **(M)** Jmjd3/UTX demethylase activity was performed using the JMJD3/UTX demethylase activity assay kit. *p < 0.0001 compared with the WT control, #p ≤ 0.0001 (HFD+anti-miR-138), #p = 0.0006 (HFD+Pro) compared with the HFD. **(N)** Diagrammatic representation of H3K27me3 binding to Tfam promoter under HFD mediated miRNA-138 action. **(O-Q)** Level of H3K27me3 and Kdm6b enrichment on the Tfam promoter in osteoblast as assessed by ChIP-qPCR. *p < 0.0001 compared with the WT control, #p ≤ 0.0001 compared with the HFD. **(R)** qRT-PCR validation of Tfam expression in osteoblast of experimental mice. *p < 0.0001 compared with the WT control, #p ≤ 0.001 compared with the HFD. All the data (Figure-2) are analyzed by one-way ANOVA followed by a Tukey's multiple comparisons test. n = 6 mice per group. All data are expressed as mean± s.e.m.

Excessive production of endotoxic LPS/endotoxin elicits exaggerated signaling through toll-like receptor 4 (Tlr4) 39. The activation of the Tlr4 signaling resulting in the inflammatory cytokine secretion in osteoblast 39. Hence, blocking the binding of LPS/endotoxin to TLR4 may reduce the osteoblast dysfunction. If the mechanism of action of IPA involves interference with Tlr4 signaling, IPA treatment of HFD group mice should cause a reduction of the osteoblast's dysfunction (Figure 2A). Therefore, we hypothesized that IPA could inhibit Tlr4 receptor expression and subsequently prevents the endotoxin mediated Tlr4 activation. To understand the IPA action on Tlr4 function in osteoblast, we have generated an osteoblast-specific ablation of Tlr4 in mice (Osx-Tlr4 KO or Tlr4^ObKO^) using Osx-cre transgenic mice crossed to Tlr4^f/f^ mice. Using western blot and mRNA expression studies, we confirmed that Tlr4 expression was indeed increased in osteoblast lysates of HFD group compared to WT control. However, the above effect was mitigated or reduced in osteoblast lysates of Tlr4^ObKO^+HFD, HFD+IPA, and HFD+Pro treatment groups (Figure 2B-D).

To study the mechanistic basis of probiotic treatment induced Tfam gene expression in the isolated osteoblast of femoral bone, we used microRNA profiling. The miRNA array (RT2 miRNA PCR Array) analysis, data demonstrated that several miRNAs were differentially expressed in HFD mice, as represented in hierarchical clustering analysis (Figure 2E). Among them, miRNA-138 was indeed upregulated in HFD group compared to the WT control (Figure 2E, black arrow). Using real-time qPCR, we further confirmed miR-138 transcript expression in the HFD group (Figure 2F). However, probiotics administration or Tlr4^ObKO^ mice fed HFD restored the miR-138 transcript expression in osteoblast of HFD+Pro or HFD+Tlr4^ObKO^ mice compared to HFD mice (Figure 2F). Further, to explore the role of miR-138 involved in the function on the histone methylation, histone remodeling marker, histone H3 lysine 27 demethylases (Kdm6b/Jmjd3) was analyzed. Through *in silico* analysis with the Targetscan program, the data demonstrated that the miR-138 sequence recognized the 45-52 bp of the conserved sequence of Kdm6b (Figure 2G). We also found a miR-22 sequence that indeed recognized the 123-129 bp of the conserved sequence of Kdm6b. However, miR-22 expression was not changed among the WT, HFD and HFD+Pro conditions (Figure S2A-B). Following *in silico* analysis, the data confirmed that mRNA transcript levels of Kdm6b were indeed reduced in HFD group, whereas its levels were improved in the HFD+Tlr4^ObKO^ or HFD+Pro condition (Figure 2H). Probiotics or IPA treatment promoted the luciferase activity of reporters containing the 3' UTR of Kdm6b, which was inhibited in HFD mice compared to WT control (Figure 2I), as assessed by dual-luciferase reporter assay. Using western blot analysis, we observed protein expression of Kdm6b/JMJD3 in differentiated osteoblast *in vitro* and data show that Kdm6b/JMJD3 was significantly reduced in HFD group compared to WT control. However, its level was enhanced in the HFD+Pro group compared to the HFD group (Figure 2J-K). To further confirm the involvement of Kdm6b function under miR-138 regulation, we depleted miR-138 expression with a specific miR-138 antagonist or anti-miR-138. Importantly, we observed that the Kdm6b/JMJD3 expression was improved when miR-138 was depleted in the HFD+anti-miR-138 group compared to HFD group (Figure 2J-K). In contrast, H3K27me3 expression was indeed increased in the HFD group compared to control and its level was reduced in HFD+pro and HFD+anti-miR-138 groups compared to HFD group (Figure 2J-L). Additionally, data also confirmed reduced enzymatic Kdm6b/JMJD3 demethylase activity in osteoblast of HFD group (Figure 2M). Administration of probiotics or anti-miR-138 reversed the changes in HFD+pro or HFD+anti-miR-138 condition.

To verify whether decreased Kdm6b expression via HFD dependent upregulation of miR-138 affects Tfam promoter expression in osteoblast, we assessed H3K27me3 enrichment and Kdm6b/JMJD3 binding levels within the Tfam promoter using a ChIP assay (Figure 2N). The data demonstrate that a striking increase of H3K27me3 enrichment (Figure 2O-P) and a significant decrease of Kdm6b/JMJD3 binding (Figure 2O, Q) were observed at the Tfam promoter in osteoblasts of HFD group compared to the WT control. However, probiotics or anti-miR-138 administration reverses the above changes. Moreover, we detected the Tfam mRNA expression in osteoblast of HFD condition using real-time PCR. Compared with the HFD group, the mRNA expressions of Tfam were significantly improved in osteoblast of HFD+Pro or HFD+anti-miR-138 group (Figure 2R). Interestingly, direct IPA supplementation significantly improved Tfam expression in the HFD+IPA group compared to the HFD group (Figure 2R). These data indicated that probiotics regulate the expression of Tfam by possibly inhibiting the miR-138 function dependent JMJD3-H3K27me3 epigenetic mechanism in osteoblasts of HFD mice.

### Probiotics promote Tfam dependent osteoblastic glycolysis and mitochondrial biogenesis via Kdm6b-Tfam axis in obese mice

**Figure 3:**
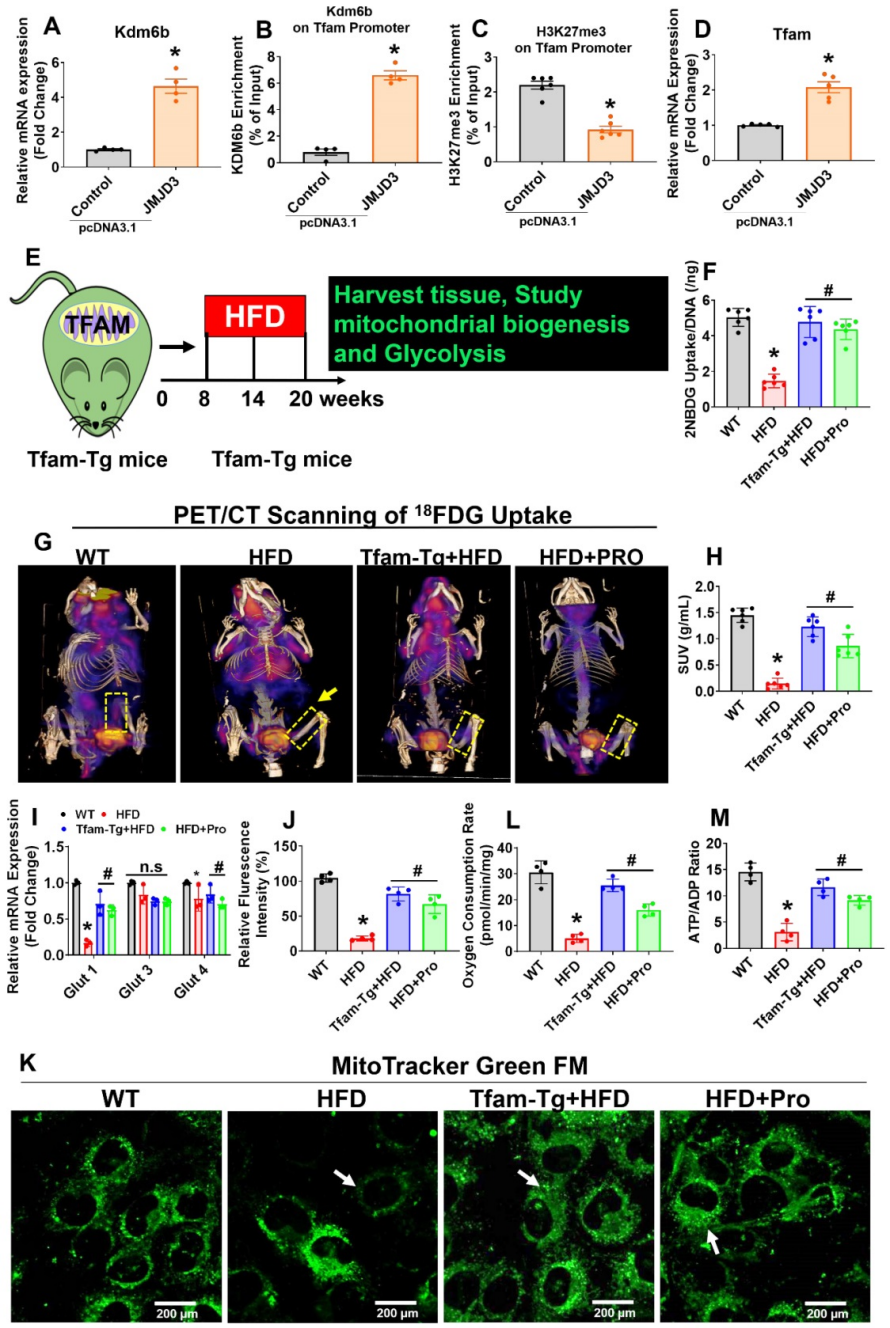
** Probiotics treatment increased glycolysis rate and mitochondrial respiration in osteoblast via TFAM overexpression in HFD fed obese mice. (A)** Osteoblasts were transfected with pcDNA3.1-JMJD3 plasmid and scramble control. Kdm6b/Jmjd3 transcript expression was confirmed by qPCR. *p = 0.0001 compared with the WT control, by unpaired t-tests. n = 3 samples per group. **(B-C)** Kdm6b and H3K27me3 enrichment on the Tfam promotor was performed in osteoblast by ChIP assay. *p < 0.0001 compared with the WT control, by unpaired t-tests. n = 3 samples per group. **(D)** Tfam transcript expression in osteoblast was studied by qPCR. *p < 0.0022 compared with the WT control, by unpaired t-tests. n = 3 samples per group. **(E)** 8-week-old Tfam-Tg mice were fed on HFD for 8-weeks. Following HFD feeding, the samples were harvested for further experiments. **(F)** 2-NBDG uptake in cultured osteoblast (normalized for DNA content of the well, n = 6). *p < 0.0001 compared with the WT control, #p< 0.0001 compared with the HFD. **(G-H)** ^18^F-FDG uptake of the femoral bone of experimental mice by micro-PET/CT Imaging. (H) [^18^F]FDG accumulations in osteoblast in bones of the experimental mice (WT, HFD, Tfam-Tg+HFD, HFD+Pro) and expressed as [18F]FDG SUVmeanvalues. *p < 0.0001 compared with the WT control, #p < 0.0001 compared with the HFD. **(I)** mRNA transcript expression of Glut1, Glut3, and Glut4 mRNA in isolated osteoblast, determined by qPCR *in vitro*. *p < 0.0001 compared with the WT control, #p < 0.0001 compared with the HFD. **(J-K)** Mitochondrial imaging was performed using confocal microscopy in the cultured osteoblast that were stained with MitoTracker™ Green FM. Scale bar: 200 µm. The bar graph represents the relative fluorescence intensity of mitochondrial contents. *p < 0.0001 compared with the WT control, #p < 0.0001 compared with the HFD. **(L)** Oxygen consumption rates measured in freshly isolated mitochondria from iolsated osteoblast using palmitoylcarnitine as a substrate. *p < 0.0001 compared with the WT control, #p ≤ 0.0001 (Tfam-Tg+HFD), #p = 0.0009 (HFD+Pro) compared with the HFD. **(M)** ATP production was measured in freshly isolated mitochondria from osteoblast. *p < 0.0001 compared with the WT control, #p ≤ 0.0001 (Tfam-Tg+HFD), #p = 0.0005 (HFD+Pro) compared with the HFD. All the data of Figure-3 were analyzed by one-way ANOVA and Figure 3i was analyzed with two-way ANOVA followed by a Tukey's multiple comparisons test. n = 6 mice per group. All data are expressed as mean± s.e.m.

Due to the decreased expression of Kdm6b and increased enrichment of H3K27me3 within the Tfam promoter, our work suggests that H3K27me3 indeed down-regulate Tfam expression through suppression of transcriptional binding of Kdm6b at Tfam promoter (Figure 2). To confirm this hypothesis, we further transfected cultured osteoblasts from HFD mice with pcDNA3.1-JMJD3/Kdm6b plasmid and estimated the consequences of H3K27me3/Kdm6b enrichment and Tfam expression. Forty-eight h after transfection, JMJD3/Kdm6b expression was significantly enhanced by pcDNA3.1-JMJD3/Kdm6b, compared with the pcDNA3.1-control plasmid (Figure 3A). Using ChIP assay, the data confirmed that JMJD3/Kdm6b binding to the Tfam promoter was also increased in the pcDNA3.1-JMJD3/Kdm6b group (Figure 3B). Consistently, H3K27me3 enrichment at the Tfam promoter was suppressed after Kdm6b up-regulation (Figure 3C). The Tfam mRNA levels were also obviously up-regulated in osteoblast transfected with pcDNA3.1-JMJD3/Kdm6b compared with the pcDNA3.1-control group (Figure 3D).

Earlier studies demonstrate that Tfam overexpression in skeletal muscle prevents unloading induced muscle atrophy 40. To determine whether overexpression of Tfam was sufficient to restore osteoblast function, we used Tfam transgenic (Tfam-Tg) mice that were fed an HFD for 8 weeks and the effects of Tfam overexpression on glycolysis rate and mitochondrial function were investigated (Figure 3E). To quantify the uptake of glucose in isolated osteoblast *in vitro*, we administered 2-NBDG, a fluorescently labeled glucose in various experimental conditions for 6 h. The glucose uptake capacity of osteoblasts was significantly reduced in HFD group compared with WT control, whereas these changes were improved in HFD+Pro, and Tfam-Tg+HFD groups compared to HFD group (Figure 3F). Interestingly, we also observed an increased uptake of glucose upon JMJD3/Kdm6b overexpression in osteoblast of HFD (HFD+JMJD3) group compared to HFD group (Figure S3A). To quantify the glucose consumption by the skeleton of experimental mice *in vivo*, we performed micro-PET/CT scans and biodistribution assays using the radioactive tracer ^18^F-fluorodeoxyglucose (^18^F-FDG) *in vivo*. The data demonstrates that the skeleton of HFD mice was found to take up a considerably smaller portion of glucose compared to the skeleton of WT mice (Figure 3G, dotted box). Strikingly, the uptake of glucose was significantly and consistently improved in the skeletons of Tfam-Tg+HFD and HFD+Pro mice compared to HFD animals, as shown by femoral bone tissue-specific quantification of the ^18^F-FDG SUVs uptake (Figure 3G-H). The data further shows that glucose transporter, such as Glut1 (albeit not Glut3 or Glut4), was increased upon overexpression of Tfam or probiotics treatment in the HFD group *in vitro* and* in vivo* (Figure 3I, Figure S3B).

To quantify the metabolic changes, we tested intracellular glucose and lactate levels in osteoblast culture of various experimental conditions (Figure S3C, D). Consistent with decreased glycolysis in the HFD condition, we tested the genes encoding the glycolysis-regulating enzymes PGK1, PDK1, and HK II. They were significantly downregulated in HFD condition compared to WT control, whereas, its levels were upregulated in osteoblast of Tfam-Tg+HFD and HFD+pro conditions compared to the HFD condition (Figure S3E). We then examined the mitochondrial contents in osteoblast culture by using a mitotracker green probe. Using confocal imaging, the data showed that mitochondrial mass was significantly reduced in HFD condition. However, these changes were improved in the osteoblast of the Tfam-transgenic (Tg)+HFD, HFD+pro in comparison to the HFD condition (white arrow, Figure 3J-K). Tricarboxylic Acid (TCS) cycle enzyme gene expression of citrate synthase (CS) and isocitrate dehydrogenase (Idh2) were restored in osteoblasts of the Tfam-Tg+HFD, HFD+Pro condition compared to HFD condition (Figure S3F). Consequently, mitochondrial oxygen consumption rates and mitochondrial ATP production were reduced in the isolated mitochondria of osteoblasts of HFD mice compared to WT control. However, these changes were improved significantly in the Tfam-Tg+HFD, and HFD+pro conditions (Figure 3L-M). Additionally, the details of metabolic parameters (body weight, average daily food intake, glucose tolerance test (GTT) and plasma insulin) of obesity in HFD fed mice under probiotics supplementation was represented in Figure S4A-F.

### Probiotics or IPA treatment restores osteoblast differentiation and mineralization *in vitro*

**Figure 4:**
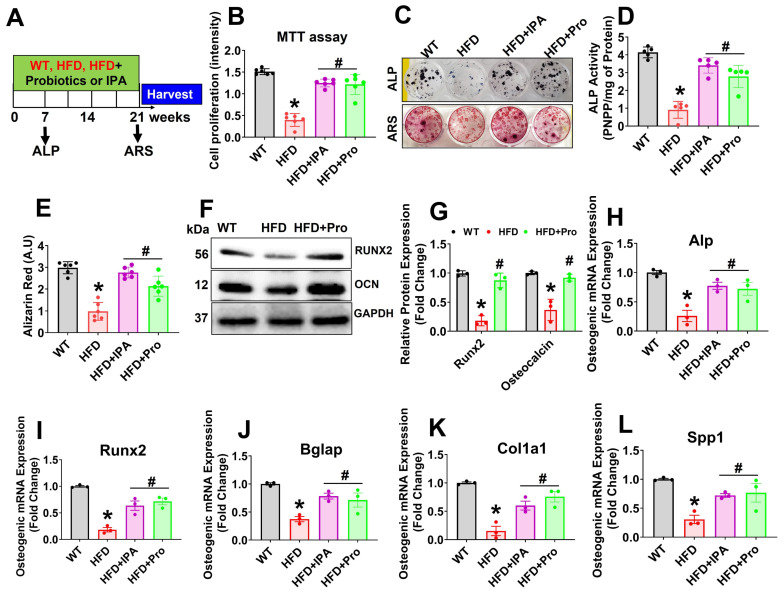
** Probiotics treatment increases osteoblast mineralization and osteogenesis *in vitro.* (A)** Experimental design and probiotics/IPA induced osteoblast differentiation and mineralization during osteogenesis. **(B)** Osteoblastic cell proliferation was measured using MTT assay. *p < 0.0001 compared with the WT control, #p ≤ 0.0001 compared with the HFD. **(C)** Effect of probiotics or IPA, on the osteoblast ALP and ARS staining was performed on day 6 and day 21. **(D)** ALP activity was measured on day 6. *p < 0.0001 compared with the WT control, #p ≤ 0.0001 compared with the HFD. **(E)** Osteoblast bone mineralization or calcium nodule assay was performed by Alizarin Red staining (ARS) on Day 21. *p < 0.0001 compared with the WT control, #p=0.0001 compared with the HFD. **(F-G)** Osteogenic protein (Runx2 and OCN) expressions were performed using western blot analysis in osteoblast culture. *p < 0.0001 compared with the WT control, #p ≤ 0.0001 compared with the HFD. **(H-L)** mRNA transcript expression of Alp, Runx2, Bglap, Cola1a, and Spp1 in osteoblast culture. *p < 0.0009 compared with the WT control, #p ≤ 0.0086 (HFD+IPA) , #p ≤ 0.0159 (HFD+Pro) for Figure 4H., *p < 0.0001 compared with the WT control, #p ≤ 0.0026 (HFD+IPA), #p ≤ 0.0009 (HFD+Pro) for Figure 4I., *p < 0.0013 compared with the WT control, #p ≤ 0.0168 (HFD+IPA), #p ≤ 0.0428 (HFD+Pro) for Figure 4J., *p < 0.0002 compared with the WT control, #p ≤ 0.0116 (HFD+IPA), #p ≤ 0.0019 (HFD+Pro) for Figure 4K., *p < 0.0027 compared with the WT control, #p ≤ 0.0472 (HFD+IPA), #p ≤ 0.0283 (HFD+Pro) for Figure 4L, compared with the HFD. All the data of Figure-4 were analyzed by one-way ANOVA followed by a Tukey's multiple comparisons test. n = 6 mice per group. All data are expressed as mean± s.e.m.

To evaluate the potential effect of probiotics or IPA in osteoblast differentiation and osteogenesis in HFD condition, we cultured isolated osteoblast in the osteogenic induction medium (OIM) for 21 days (Figure 4A). Interestingly, data suggests that cell proliferation was reduced in HFD condition on day 3 compared to WT control. However, these changes were improved in HFD+IPA and HFD+Pro condition compared to HFD condition (Figure 4B). We also found that osteoblast exhibited an increase in alkaline phosphatase (ALP) activity and staining on Day 6 in the HFD+IPA and HFD+Pro condition compared to HFD condition (Upper panel, Figure 4C-D). Alizarin red staining (ARS) also confirmed that there was reduced mineralized nodule formation on Day 21 compared to WT control. However, these changes were improved in the HFD+IPA and HFD+Pro conditions compared to the HFD condition (Lower panel, Figure 4C, E). Additionally, increased osteoblastic mineralized nodule formation was observed in the HFD+Tlr4^ObKO^, HFD+anti-miR-138, HFD+JMJD3 and Tfam-Tg+HFD condition compared to HFD condition alone respectively (Figure S5A-B). Similarly, Western blot analysis further confirmed the expression of osteogenesis-related proteins, such as Runx2 and osteocalcin (OCN), significantly reduced in HFD condition, whereas these changes were improved in the HFD+Pro condition compared to HFD condition (Figure 4F-G). Further, the data also demonstrated that the mRNA transcripts of osteogenic genes Alp, Runx2, Bglap, Col1a1, and Spp1 were significantly down regulated in HFD condition on Day 21. However, probiotics or IPA administration improved above changes in osteoblasts of the HFD+Pro or HFD+IPA condition compared to the HFD condition (Figure 4H-L). Thus, probiotics or IPA administration prevents HFD induced obesity associated reduction of osteogenesis and mineralization *in vitro* (Figure 4).

### Probiotics or IPA treatment prevents osteoclast differentiation and maturation *in vitro*

**Figure 5:**
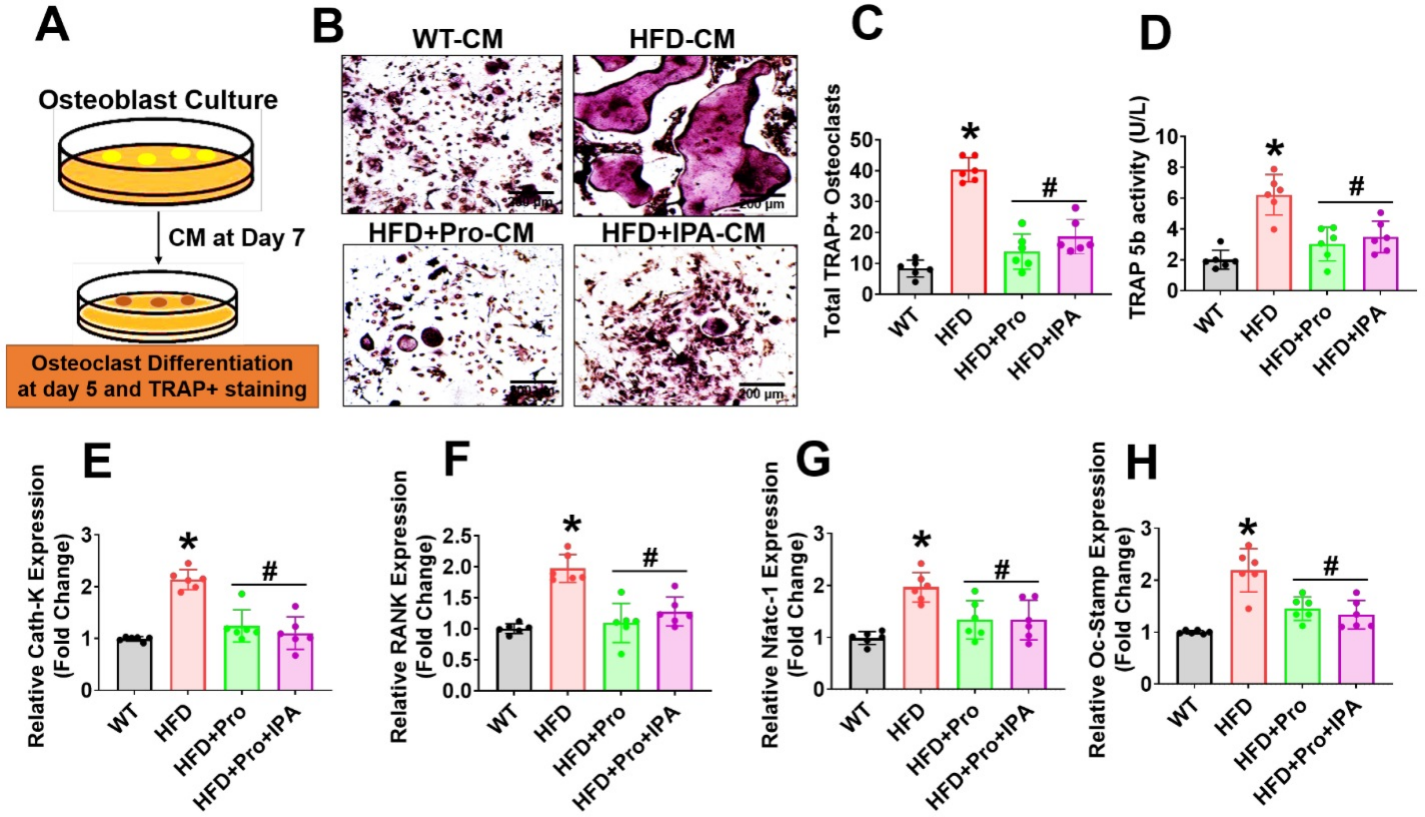
**Probiotics treatment prevents osteoclast differentiation and bone resorption *ex vivo.* (A)** Experimental strategy for induction of osteoclast differentiation and maturation following treatment with CM derived from osteoblast culture. **(B-C)** CM collected from the various experimental groups of osteoblast culture and incubated with osteoclast culture. On day 5, osteoclasts were TRAP-stained and counted the total number of TRAP+ osteoclasts in various experimental conditions. *p < 0.0001 compared with the WT control, #p ≤ 0.0001 compared with the HFD. *Scale bar: 200 µm.* **(D)** The TRAP-5b activity was measured in the differentiated osteoclast cell lysate, as assessed by ELISA. *p < 0.0001 compared with the WT control, #p ≤ 0.0002 (HFD+Pro), #p ≤ 0.0011 (HFD+IPA), compared with the HFD. **(E-H)** qPCR analysis of osteoclastic gene expression of the indicated genes: Ctsk, RANK, Nfatc1 and Oc-Stamp. *p < 0.0001 compared with the WT control, #p < 0.0001 for Figure 5E., *p < 0.0001 compared with the WT control, #p < 0.0001 (HFD+Pro), #p ≤ 0.0002 (HFD+IPA) for Figure 5F., *p ≤ 0.0001 compared with the WT control, #p < 0.0103 (HFD+Pro), #p ≤ 0.0103 (HFD+IPA) for Figure 5G., *p < 0.0001 compared with the WT control, #p < 0.0008 (HFD+Pro), #p ≤ 0.0002 (HFD+IPA) for Figure 5H. All the data of Figure-5 were analyzed by one-way ANOVA followed by a Tukey's multiple comparisons test. n = 6 mice per group. All data are expressed as mean± s.e.m.

To test whether the secretome of osteoblast from HFD condition influenced osteoclastogenesis, we performed *ex vivo* osteoclastogenesis assays by using CM derived from osteoblast culture on Day 7 (Figure 5A). The results demonstrated that HFD osteoblast culture-derived CM (HFD-CM) treated osteoclast culture had more mature, tartrate-resistant acid phosphatase-positive (TRAP+) osteoclasts by Day 5 of differentiation, compared to CM from WT osteoblast (WT-CM). However, the total number of TRAP+ osteoclasts was decreased in the HFD+IPA-CM and HFD+Pro-CM condition (Figure 5B-C). Indeed, *in vitro* TRAP 5b activity was also significantly increased in HFD-CM condition compared to WT-CM condition. However, these changes were mitigated in the HFD+IPA-CM and HFD+Pro-CM condition compared to HFD-CM condition (Figure 5D). We also tested the expression of a series of osteoclast genes such as Ctsk, RANK, Nfatc1 and Oc-Stamp, in cultured osteoclasts *in vitro*. The results showed that these genes are markedly upregulated in HFD condition in compared to WT control condition. However, these changes were significantly downregulated in the osteoclasts that were cultured under HFD+Pro-CM or HFD+IPA-CM conditions in comparison with HFD-CM. (Figure 5E-H). These results suggest that osteoblast derived CM from HFD condition which eventually exaggerated osteoclast differentiation and maturation. Interestingly, probiotics or IPA treatment reversed the HFD mediated osteoclastogenesis *in vitro*.

### Probiotics or IPA treatment prevented obese-induced bone loss phenotype *in vivo*

**Figure 6:**
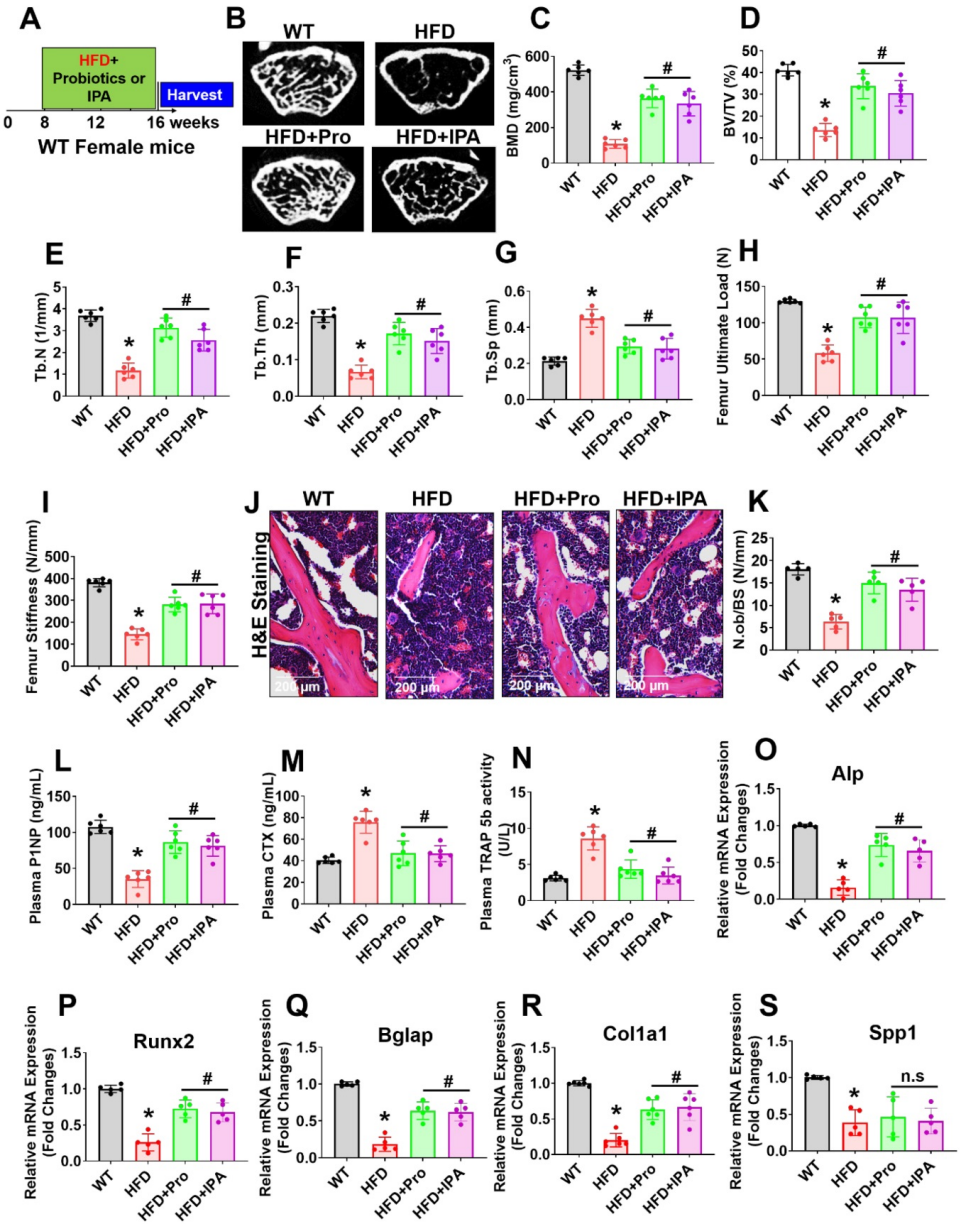
**Probiotics treatment prevented obesity-induced Osteopenia phenotype in mice. (A)** 8-weeks-old C57BL/6 mice were orally supplemented with probiotics in the presence/absence of a HFD for periods of 8-weeks. Following treatments, the bone samples were harvested for further experiments. **(B)** microCT scan of trabecular bone from the distal femur of WT, HFD, HFD+Pro, and HFD+IPA mice was performed. Scale bar, 100 µm.** (C-G)** Trabecular bone phenotype parameters were observed: Bone mineral density (BMD), Bone volume per tissue volume (BV/TV) (%), trabecular number (Tb.N (1/mm)), trabecular thickness (Tb.Th) (mm), trabecular space (Tb.Sp) (mm) from experimental mice groups. *p < 0.0001 compared with the WT control, #p ≤ 0.0001 compared with the HFD. **(H-I)** Bone biomechanical properties (Maximum load (N) and stiffness (N/mm)) in the femur of experimental mice groups. *p < 0.0001 compared with the WT control, #p ≤ 0.0001 compared with the HFD. **(J-K)** Hematoxylin and Eosin (H & E) staining of femur trabecular bone volume and black arrow indicates H&E-stained osteoblasts and expressed as the number of osteoblasts (N.Ob) per millimeter of trabecular bone surface (BS). Scale bar, 200 µm. *p < 0.0001 compared with the WT control, #p < 0.0001 (HFD+Pro), #p < 0.0002 (HFD+IPA), compared with the HFD. **(L-M)** ELISA analysis of bone formation marker, N-terminal propeptide (PINP), and bone resorption marker, CTX-I was examined in the plasma of experimental mice. *p < 0.0001 compared with the WT control, #p ≤ 0.0001 compared with the HFD. **(N)** TRAP 5b activity was assessed in the plasma of experimental mice by ELISA. *p < 0.0001 compared with the WT control, #p ≤ 0.0001 compared with the HFD. **(O-S)** mRNA transcripts expression of osteogenic genes (Alpl, Runx2, Bglap, Col1a1, and Spp1) was performed in the femoral bone tissue. *p < 0.0001 compared with the WT control and #p < 0.0001 compared with the HFD (Figure 6O-R)., *p < 0.0004 compared with the WT control, n.s (p < 0.9070, sp < 9975) compared with the HFD (Figure 6S). All the data of Figure-6 were analyzed by one-way ANOVA followed by a Tukey's multiple comparisons test. n = 6 mice per group. All data are expressed as mean± s.e.m.

To examine the role of probiotics supplementation in the promotion of skeletal homeostasis *in vivo*, mice were supplemented by oral gavage with 1×10^9^ CFU probiotics (VSL#3) or IPA (20 mg/kg/day) or vehicle control to HFD fed mice for 8 weeks (Figure 6A). Using microCT scans, the bone trabecular microarchitecture parameters (bone mineral density (BMD), bone volume to tissue volume (BV/TV) ratio, trabecular number (Tb.N), trabecular thickness (Tb.Th), and trabecular spacing (Tb.Sp)) in the femur were analyzed (Figure 6B-G). Briefly, there was a significant decrease in distal femur BMD, BV/TV ratio, Tb.N, and Tb.Th in HFD mice compared to that of WT control mice (Figure 6C-F). Furthermore, Tb.Sp was increased in HFD mice compared to the WT control group (Figure 6G), indicating an osteoporotic phenotype was observed. However, probiotics or IPA administration prevented the obese induced bone loss in the HFD+Pro or HFD+IPA condition (Figure 6B-G). In addition to this, we demonstrated the biomechanical properties of the femoral bone of experimental mice using 3-point bending tests. The data showed that the biomechanical properties, such as maximum load and stiffness, were reduced in HFD mice compare to WT control mice (Figure 6H-I). However, the above parameters were significantly improved in HFD+Pro or HFD+IPA mice (Figure 6H-I).

Analysis of femoral trabecular bone by histomorphometry revealed that probiotics and IPA administration increased the indices of bone formation such as osteoblast number (N.Ob/BS) in HFD+Pro or HFD+IPA mice in comparison to HFD mice alone (Figure 6J-K). Indices of bone turnover markers (P1NP, a marker of bone formation and CTX, a marker of bone resorption) were affected by probiotics and IPA treatment in HFD groups (Figure 6L-M). Plasma P1NP was increased by probiotics and IPA in HFD mice (Figure 6L). Plasma CTX, was decreased in response to probiotics and IPA supplementation, confirming the capacity of gut microbiota-derived IPA following probiotics or IPA supplementation to suppress bone resorption in HFD+Pro or HFD+IPA mice compared to HFD mice (Figure 6M). Besides, plasma TRAP-5b activity was significantly increased in HFD mice compared to WT control. However, these changes were improved following probiotics and IPA supplementation in HFD+Pro or HFD+IPA mice respectively compared to HFD mice (Figure 6N). Consistent with the bone formation activity of probiotics and IPA, we also tested the expression profile of various osteogenic genes in the femoral bone tissue of experimental mice *in vivo*. The data confirmed that the osteogenic mRNA transcripts of Alp, Runx2, Bglap, Col1a1, and Spp1 were reduced in HFD condition compared to WT control. However, above changes were improved in HFD+Pro or HFD+IPA conditions compared to HFD condition (Figure 6O-S). These data demonstrate that probiotics supplementation enhances bone formation by increasing the osteoblasts in the bones of HFD+Pro mice via an IPA dependent manner.

## Discussion

In the present study, we demonstrated that oral supplementation of probiotics (VSL#3) or IPA to HFD fed obese mice increased trabecular bone formation, and improved cortical bone mechanical strength. Probiotics supplementation increased the fecal levels of tryptophan metabolite, IPA in the gut as well as plasma, by manipulating the gut microbiota and increasing IPA synthesizing microbes. This critical IPA promoted the osteoblast differentiation by enhancing mitochondrial transcription activator Tfam via increased binding of histone demethylase Kdm6b and decreased binding of H3K27me3 to the Tfam promoter. The increased Tfam expression in mitochondria of osteoblasts improves mitochondrial bioenergetic metabolism and glycolysis rate. Interestingly, this leads to enhanced osteogenesis and bone formation. This discovery provided, for the first time, proof that miR-138-histone remodeling (H3K27me3) signaling is governed during the HFD induced obesity model. This instigates bone loss and lower mechanical strength, whereas administration of probiotics, or IPA, may have a therapeutic effect to enhance bone anabolism in the obese model via the gut-bone axis.

Several studies have demonstrated the role of gut microbiota on bone development studying germ-free or antibiotic-treated or pathogen-free or ovariectomy mouse model 10, 33, 41. However, understanding the role of gut microbiota during HFD induced obesity and its role in bone loss was not investigated. In the present study, we focused on two independent experimental approaches (direct supplementation of probiotics, and IPA) and discovered consistent beneficial effects in obesity-induced mitochondrial dysfunction mediated bone loss which was highly dependent on microbial-derived increased tryptophan metabolites (IPA) levels.

The recent study reported that an HFD is negatively modulated in the gut microbiota, metabolic phenotypes, and intestinal barrier function 42-44. The intestinal barrier integrity also plays a vital role in sustaining metabolic balance by thwarting the transport of harmful toxins and biomolecules 45. Therefore, we hypothesize that the manipulation of gut microbiota through probiotics could improve inflammation and intestinal permeability in an HFD induced obesity. In the current study, data revealed that the fecal microbiota composition of probiotics treated HFD+Pro mice was significantly different from HFD mice. Interestingly, we provide evidence of increased colonization and relative abundance of IPA-producing *Clostridia* in HFD+Pro mice. IPA levels among other tryptophan metabolites (IA, I3A, 5-HIAA) were significantly increased in probiotics treated HFD mice. However, gut microbiome-derived IPA that exhibits beneficial metabolic effects on gut homeostasis and bone development in the obesity model is still not understood 46, 47. In agreement with this result, we found that probiotics or IPA administration directly, exerted anabolic effects and found significantly decreased body weight gain and enhanced insulin sensitivity in HFD+Pro or HFD+IPA mice. Additionally, it is also evidently shown that gut inflammation, colonic tissue epithelial damage, and intestinal permeability including tight junction proteins (ZO-1, Occludin and Claudin-5) expression, were indeed improved upon probiotics and IPA supplementation in HFD mice.

The link between gut microbiome and bone formation was established in the estrogen deficiency (OVX) mouse model under the action of short-chain fatty acids (SCFAs) 31. However, gut microbiota-derived tryptophan metabolite, IPA and its novel function in mitochondrial biogenesis and bone development has not yet been studied. In this context, we focused on determining the nature of osteoblast activity in bone milieu following gut inflammation and how probiotics derived tryptophan metabolite, IPA or direct IPA supplementation helps promote bone anabolism in an obese mouse model. Interestingly, the data also demonstrate for the first time that IPA could inhibit the Tlr4 receptor expression and prevent the endotoxin induced osteoblast dysfunctions. We also found that probiotics increased the osteoblastic activity by downregulating miRNA-138 that regulates the aberrant H3K27me3 methylation mark within the promoter region of Tfam. Histone modifications are significant regulators of epigenetic chromatin remodeling and are found to activate gene transcription 48 effectively. Histone 3 lysine 27 methylation 3 (H3K27me3) is one of the critical posttranslational modifications of histones. It can be catalyzed to mono- (H3K27me1), di-(H3K27me2) and tri- (H3K27me3) methylated states. The mono-methylation of H3K27 (H3K27me) is associated with switching on or gene transcription, whereas tri-methylation of H3K27 (H3K27me3) is associated with switching off or gene repression 49. The results of the present study indicated that H3K27me3 levels within the Tfam promoter region of osteoblast were significantly decreased; H3K27me3 demethylase Kdm6b enrichment within Tfam promoter was increased due to low expression of miR-138 upon probiotics treatment in HFD mice. The data also found that probiotics, or IPA, supplementation was positively correlated with Tfam mRNA expression.

Bone marrow is also the primary site for hematopoiesis through the complex interplay of cell-cell communication between hematopoietic stem cells (HSCs) and their niche 50. On the other hand, HSCs undergo a series of cellular signaling and differentiate into osteoclast precursor cells (OCs), a process called as osteoclastogenesis 51. Later, the OCs are involved in bone loss by resorbing bone from the matrix 51. However, both cellular processes are altered by the introduction of HFD in mice 52. A recent study showed that HFD induced obese in mice have increased hematopoiesis (the composition of lymphocytes, monocytes, granulocytes, erythrocytes, and mixed progenitor lineages), leading to deregulated immune function 53. As a previous study found increased monocytes and macrophages are indeed altered 53 and could be fore-front precursors for inducing osteoclastogenesis in HFD-induced obese model 54. Considering, in the current study, we demonstrated that HFD is associated with increased osteoclast differentiation and TRAP-5b activity level compared to WT control *in vitro* and bone loss *in vivo*. However, future research is required to address the deleterious effect of HFD on crosstalk between hematopoiesis and osteoclastogenesis that synergistically governs obesity associated osteoporotic bone loss.

Several studies have shown that Tfam promoter DNA methylation was associated with obstructive pulmonary disease, insulin resistance, and cardiovascular disease by altering mitochondrial function and energy metabolism 55, 56. The study also found that osteoblast differentiation was negatively affected with mitochondrial stress in mouse models of SOD2 knockout mice 57. In this study, we demonstrated increased Tfam promoter histone methylation was associated with reduced levels of mitochondria and led to altered mitochondrial bioenergetics and reduced glycolysis rate. Using TFAM-Tg mice, the data demonstrate that glycolysis rate and mitochondrial biogenesis were restored in osteoblast of the TFAM-Tg+HFD condition. Additionally, overexpression of Tfam further instigates osteoblast differentiation and mineralization. Using µCT scans, we observed the pivotal role of probiotics that affect bone formation *in vivo* through modulation of microbial-derived IPA. Our data revealed that probiotics and IPA did induce bone anabolism in HFD mice by improving mitochondrial Tfam function in osteoblast, demonstrating that the effects of probiotics and IPA were mediated through the promotion of active energy metabolism in osteoblast. Further, cortical bone mechanical strength was improved following probiotics or IPA treatment.

Therefore, it could be proposed that probiotics act as a biological active energy modulator via nutritional supplementation to increase the osteoblast activity in the skeletal system. Here, we demonstrated novel mechanistic data that probiotics maintain gut microbiota by especially promoting IPA synthesizing microbes, preventing intestinal inflammation and permeability. Further probiotics improves Tfam dependent mitochondrial biogenesis, and osteoblast function by inhibiting the Tlr4/miR-138-H3K27me3 axis. Mechanistic studies revealed that, Kdm6b binds to the Tfam promoter and encourages its expression. This exaggerated expression of Tfam maintain mitochondrial bioenergetics and glycolysis rate and encourages osteoblast mineralization and bone mass. Overall, probiotics, or IPA, increases bone anabolism via the gut-bone axis. Therefore, our work provides evidence that probiotics or IPA may find broader applications, as a treatment for inflammatory, and metabolic osteoporosis conditions in future translational medicine.

## Supplementary Material

Supplementary figures and tables.
