# Supplementary material for: Probiotics Stimulate Bone Formation in Obese Mice via Histone Methylations
Source: Theranostics. 2021 Jul 25;11(17):8605–23. doi: 10.7150/thno.63749 (PMC8344023; doi:10.7150/thno.63749)
Supplement: Supplementary file 1 — Supplementary figures and tables. [file thnov11p8605s1.pdf]

Supplemental Information

Probiotics Stimulate Bone Formation in Obese Mice via Histone Methylations

Jyotirmaya Behera[1], Jessica Ison[1], Michael J. Voor[2, 3], Neetu Tyagi[1*]

[1]Bone Biology Laboratory, Department of Physiology, School of Medicine, University of Louisville, Louisville, KY 40202 USA

[2]Department of Orthopaedic Surgery, School of Medicine, University of Louisville, Louisville, KY 40202 USA

[3]Department of Bioengineering, Speed School of Engineering, University of Louisville, Louisville, KY 40202 USA

Running title: Gut Microbiota Manipulation Promotes Bone Formation in Obese Mice via Histone Methylations

***Address for Correspondence:**

Neetu Tyagi, Ph.D., FAPS
Associate Professor
Bone Biology Laboratory
Department of Physiology
Health Sciences Centre, A-1201,
University of Louisville
Louisville, KY 40202
Phone: 502-852-4145
Fax: 502-852-6239
E-mail: n0tyag01@louisville.edu

Supplementary Figure.

Supplementary Figure S1

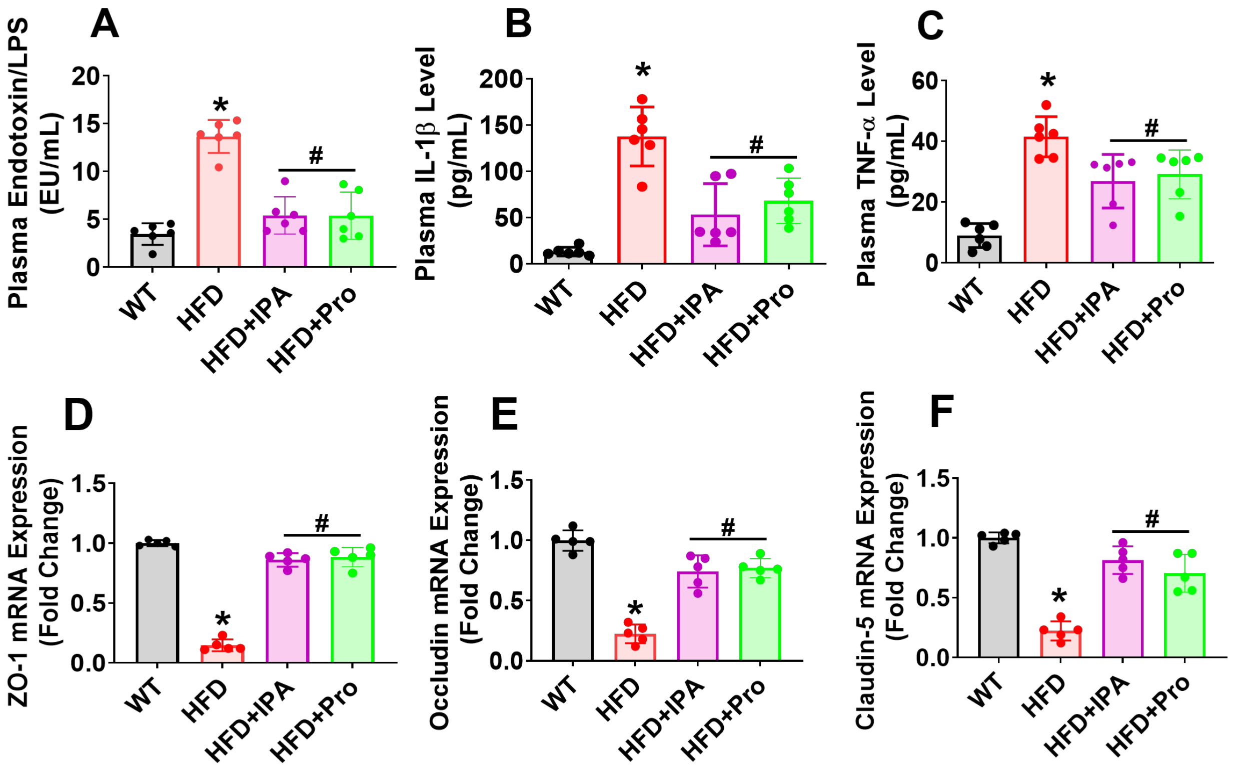

Supplementary Figure S1. Effect of probiotics or IPA treatment on endotoxin and inflammatory cytokine level in HFD mice. **(A)** The plasma endotoxin level was detected using the Limulus Amebocyte Lysate kit. *p < 0.0001 compared with the WT control, #p < 0.0001 compared with the HFD, by one-way ANOVA followed by a Tukey's multiple comparisons tests. **(B)** Plasma IL-1β levels were measured using ELISA. *p < 0.0001 compared with the WT control, $^{\#}$p < 0.0001 (HFD+IPA), $^{\#}$p < 0.001 (HFD+Pro) compared with the HFD, by one-way ANOVA followed by a Tukey's multiple comparisons test. **(C)** Plasma TNF-α level was measured using ELISA. *p < 0.0001 compared with the WT control, $^{\#}$p < 0.0095 (HFD+IPA), $^{\#}$p < 0.0318 (HFD+Pro) compared with the HFD. **(D-F)** qPCR analysis of mRNA transcript expression of tight junctions' proteins (ZO-1, Occludin, and Claudin-5) in the experimental group. *p < 0.0001 compared with the WT control, #p < 0.0001 compared with the HFD for fig. S1D-F, by one-way ANOVA, followed by a Tukey's multiple comparisons test. All data are expressed as mean± s.e.m. n = 5-6 mice per group. The error bars represent the s.e.m.

Supplementary Figure S2

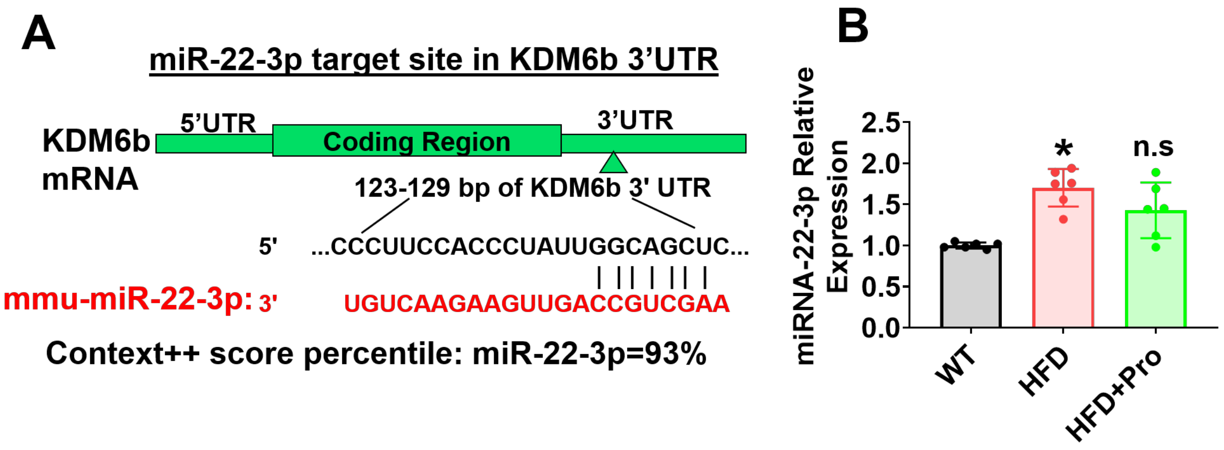

Supplementary Figure S2. The prediction of the miR-22 binding site and its expression. (a) Schematic representation of the miR-22-3p binding sites in histone demethylase, Kdm6b/Jmjd3. **(b)** miR-22-3p expression in osteoblast. $^{*}p < 0.0003$ compared with the WT control and $^{n.s}p < 0.1458$ compared with the HFD by one-way ANOVA followed by a Tukey's multiple comparisons test. n = 6 mice per group. All data are expressed as mean± s.e.m. The error bars represent the s.e.m.

Supplementary Figure S3

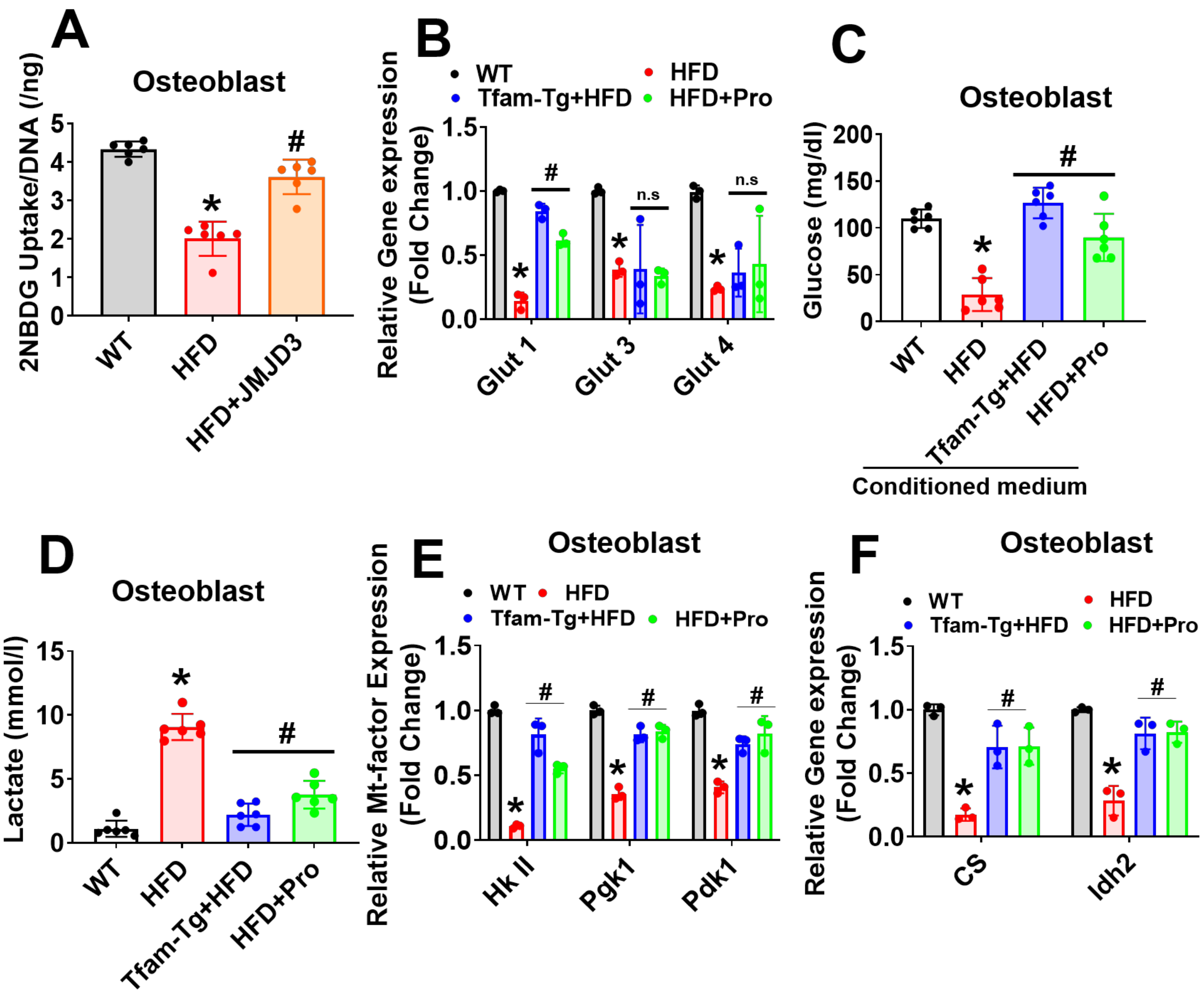

Supplementary Figure S3. Effect of probiotics on osteoblast and glycolytic response. (a) 2-NBDG uptake in cultured osteoblast upon JMJD3/Kdm6b overexpression (normalized for DNA content of the well, n = 6). *p < 0.0001 compared with the WT control, #p< 0.0001 compared with the HFD. **(b)** mRNA transcript expression of Glut 1, 3, 4 expression in femoral tissue of various experimental conditions. *p < 0.0001 compared with the WT control and #p < 0.0109 (HFD+Pro), #p < 0.0001 (Tfam-Tg+HFD) compared with the HFD by one-way ANOVA followed by a Tukey's multiple comparisons test. N.s denotes not significant. **(c-d)** Glucose and lactate level was estimated in osteoblast lysate and culture supernatants, respectively. *p < 0.0001 compared with the WT control and #p < 0.0001 compared with the HFD by one-way ANOVA followed by a Tukey's multiple comparisons test. n = 6 mice per group. **(e)** mRNA transcript expression of glycolytic enzymes (Hk II, Pgk 1, Pdk 1) by qPCR assay. *p < 0.0001 compared with the WT control and #p < 0.0001 compared with the HFD by one-way ANOVA followed by a

Tukey's multiple comparisons test. **(f)** TCA cycle enzyme Cs and Idh2 mRNA transcript expression were analyzed by the qPCR assay. *p < 0.0001 compared with the WT control and #p < 0.0001 compared with the HFD by one-way ANOVA followed by a Tukey's multiple comparisons test. All data are expressed as mean± s.e.m. n = 3 mice per group. The error bars represent the s.e.m.

Supplementary Figure S4

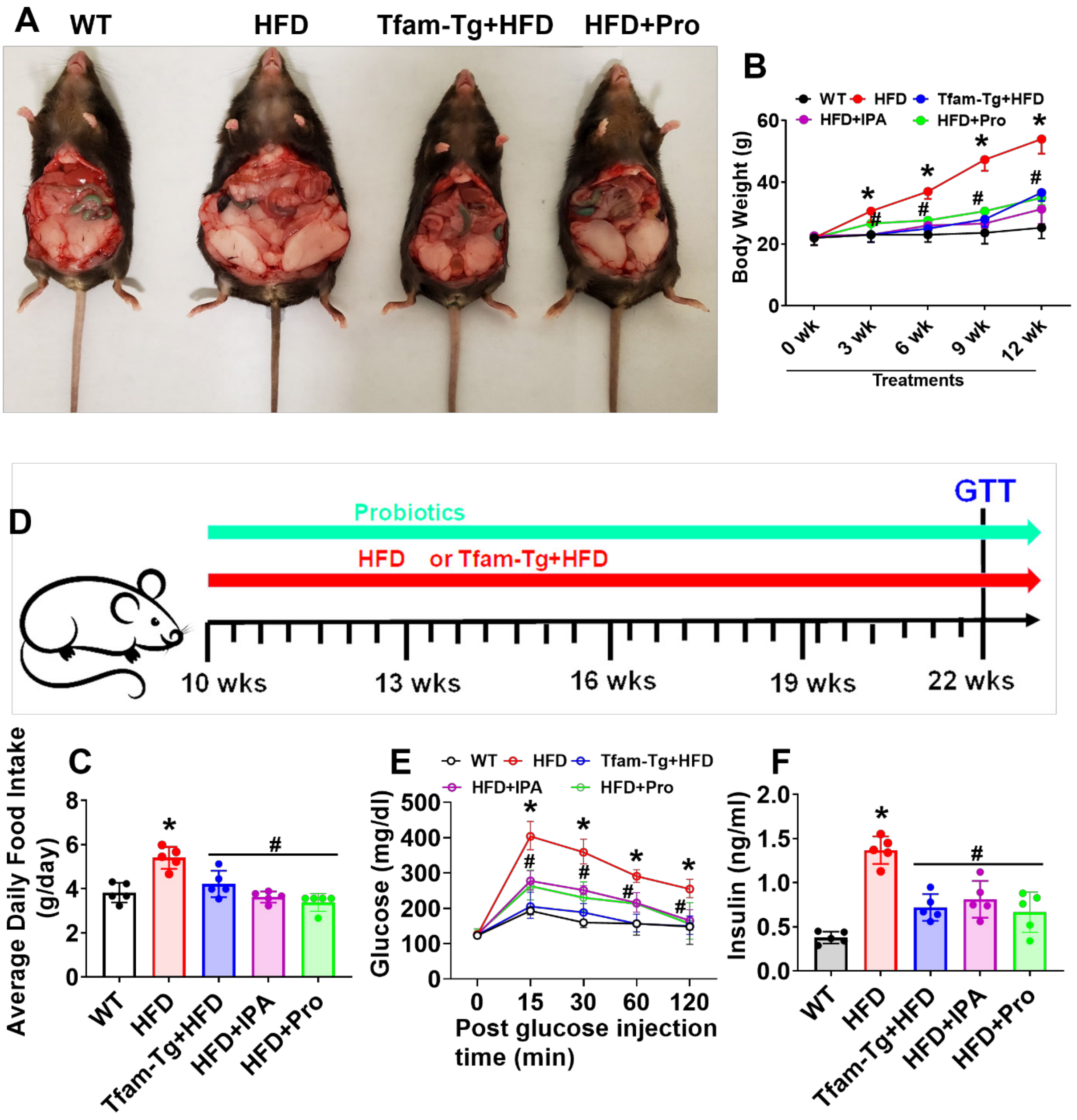

Supplementary Figure S4. Tfam overexpression or probiotics supplementation prevents HFD-induced obesity and impaired glucose tolerance. (a) Photograph of representative mice of various experimental conditions. **(b)** Bodyweight monitored throughout the treatments (starting from 0 wks to 12 wks). *p < 0.001 compared with the WT control and #p < 0.001 compared with the HFD by two-way ANOVA followed by a Tukey's multiple comparisons test. **(c)** Average daily food intake by mice during 8 weeks of treatments. *p < 0.0002 compared with

the WT control and #p < 0.0001 (HFD+IPA or HFD+Pro), #p < 0.0041 (Tfam-Tg+HFD) compared with the HFD by one-way ANOVA followed by a Tukey's multiple comparisons test. **(d-e)** Glucose tolerance test (GTT) after 8 weeks of diet or probiotics or IPA supplementation by using an i.p. the dose of 1 g of glucose per kg of body weight. *p < 0.01 compared with the WT control and #p < 0.01 compared with the HFD by two-way ANOVA followed by a Tukey's multiple comparisons test. **(f)** Plasma insulin level was measured in the experimental mice. *p < 0.0001 compared with the WT control and #p < 0.0001 (Tfam-Tg+HFD), #p = 0.0004 (HFD+IPA) compared with the HFD by one-way ANOVA followed by a Tukey's multiple comparisons test. All data are expressed as mean± s.e.m. The error bars represent the s.e.m.

Supplementary Figure S5

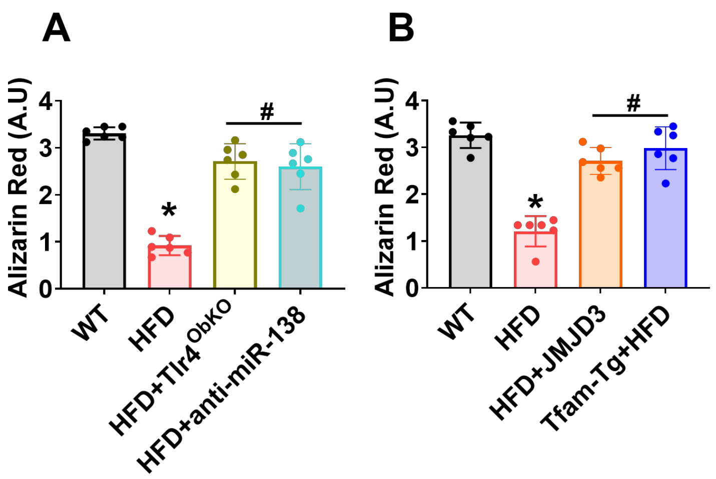

Supplementary Figure S5. Restoring the osteoblast mineralization using Tfam overexpression. (a) Effect of Tlr4 knockout and miR-138 inhibition (anti-miR-138) on Osteoblast mineralization, were performed using alizarin red assay (ARS). *p < 0.0001 compared with the WT control and #p < 0.0001 compared with the HFD by two-way ANOVA followed by a Tukey's multiple comparisons test. All data are expressed as mean± s.e.m. **(b)** Effect of JMJD3 and Tfam overexpression on Osteoblast mineralization was demonstrated using ARS assay. *p < 0.0001 compared with the WT control and #p < 0.0001 compared with the HFD by one-way ANOVA followed by a Tukey's multiple comparisons test. All data are expressed as mean± s.e.m. The error bars represent the s.e.m.

Supplementary Table S1: Sequences of primers used for real-time quantitative PCR

Gene Name	Primer sequence 5'-3'
Runx2	FP: TTTAGGGCGCATTCCTCATC<br>RP: TGTCCTTGTGGATTGAAAGGAC
Bglap	FP:GCGCTCTGTCTCTCTGACCT<br>RP: ACCTTATTGCCCTCCTGCTT
Alpl	FP: CCAGAAAGACACCTTGACTGTGG<br>RP: TCTTGTCCGTGTCGCTCACCAT
Col1a1	FP: CCTCAGGGTATTGCTGGACAAC<br>RP: CAGAAGGACCTTGTTTGCCAGG
Spp1	FP: GACAACAACGGAAAGGGCAG<br>RP: GATCGGCACTCTCCTGGCT
Ctsb	FP:GGATGAAATCTCTCGGCGTTT<br>RP:GGTTATGGGCAGAGATTTGCTT
Nfatc1	FP:GAGACAGACATCCGGAGGAAGA<br>RP:GTGGGATGTGAACACGGAAGA
RANK	FP:ACTGAGGAGGCCACCCAAGGA<br>RP:TGAAGAGGACCAGAACGATGAG
Hk 2	FP: TGATCGCCTGCTTATTCACGG<br>RP: AACCGCCTAGAAATCTCCAGA
Pdk1	FP: CCCCGATTCAGGTTCACG<br>RP: CCCGGTCACTCATCTTCACA
Pgk1	FP: GGAGCGGGTCGTGATGA<br>RP: GCCTTGATCCTTTGGTTGTTTG
Glut 4	FP: CCAGCCACGTTGCATTGTA<br>RP: ACACTGGTCCTAGCTGTATTCT
Glut 3	FP:TGGTAGCTCAGATCTTTGGTTTGG<br>RP:GATCTCTGTAGCTTGGTCTTCCTC
Glut 1	FP: GGGCATGTGCTTCCAGTATGT<br>RP: ACGAGGAGCACCGTGAAGAT
mtTFA	FP: GGAGGCAAAGGATGATTCGG<br>RP: TCGTCCAACTTCAGCCATCT
Sdha	FP: GAGATACGCACCTGTTGCCAAG<br>RP: GGTAGACGTGATCTTTCTCAGGG
GAPDH	FP: TGCACCACCAACTGCTTGC<br>RP: GGCATGGACTGTAGTCAGAG
miRNA-138-3p	FP: AGCTGGTGTTGTGAATCAGGCCG<br>RP: GCCTGATTCACAACACCAGCTTT
U6snRNA	FP:CTCGCTTCGGCAGCACA<br>RP:AACGCTTCACGAATTTGCGT
**ChIP Primers**	
mtTFA	FP:CAGTCCATAGGCACCGTATTG<br>RP:CAAGGCAGAAGGAGAGCG

Footnotes: Runt-related transcription factor 2 (RUNX2), bone gamma-carboxyglutamic acid-containing protein (Bglap), Mitochondrial transcription factor A (mtTFA or Tfam).

Supplementary Table S2: Primary antibodies and mitochondrial specific probes used

**Antibody**	**Clone**	**Dilution**	**Source**	**Cat. No**
**Western blot**				
Rabbit Anti-RUNX2 antibody	polyclonal	1:250	Abcam	ab23981
Rabbit Anti-Osteocalcin antibody	polyclonal	1:250	Abcam	ab93876
Rabbit anti-Kdm6b/Jmjd3 Antibody	polyclonal	1:500	Abcam	ab169197
Rabbit anti-Tfam Antibody	polyclonal	1:250	Abcam	ab131607
Mouse anti-GAPDH Antibody	Monoclonal, clone 6C5	1:500	Millipore	CB1001
Mouse anti-H3K27me3 Antibody	Monoclonal, ChIP grade	1:250	Abcam	ab6002
Rabbit anti-H3 Antibody	polyclonal	1:500	Abcam	ab18521
**Flow cytometry and immunofluorescence**	**Indicator**	**Dilution**	**Source**	**Cat. No**
MitoTracker™ Green FM	Mitochondria imaging	100 nM	Invitrogen Molecular Probes	M7514
